# Probing of G-Quadruplex Structures via Ligand-Sensitized Photochemical Reactions in ^Br^U-Substituted DNA

**DOI:** 10.1038/s41598-018-34141-z

**Published:** 2018-10-25

**Authors:** Abhijit Saha, Sophie Bombard, Anton Granzhan, Marie-Paule Teulade-Fichou

**Affiliations:** 1 CNRS UMR9187, INSERM U1196, Institut Curie, PSL Research University, 91405 Orsay, France; 2 CNRS UMR9187, INSERM U1196, Université Paris Sud, Université Paris Saclay, 91405 Orsay, France

## Abstract

We studied photochemical reactions of ^Br^U-substituted G-quadruplex (G4) DNA substrates with two pyrene-substituted polyazamacrocyclic ligands, M-1PY and M-2PY. Both ligands bind to and stabilize G4-DNA structures without altering their folding topology, as demonstrated by FRET-melting experiments, fluorimetric titrations and CD spectroscopy. Notably, the bis-pyrene derivative (M-2PY) behaves as a significantly more affine and selective G4 ligand, compared with its mono-pyrene counterpart (M-1PY) and control compounds. Upon short UVA irradiation (365 nm) both ligands, in particular M-2PY, efficiently sensitize photoreactions at ^Br^U residues incorporated in G4 structures and give rise to two kinds of photoproducts, namely DNA strand cleavage and covalent ligand–DNA photoadducts. Remarkably, the photoinduced strand cleavage is observed exclusively with G4 structures presenting ^Br^U residues in lateral or diagonal loops, but not with parallel G4-DNA structures presenting only propeller loops. In contrast, the formation of fluorescent photoadducts is observed with all ^Br^U-substituted G4-DNA substrates, with M-2PY giving significantly higher yields (up to 27%) than M-1PY. Both ligand-sensitized photoreactions are specific to ^Br^U-modified G4-DNA structures with respect to double-stranded or stem-loop substrates. Thus, ligand-sensitized photoreactions with ^Br^U-substituted G4-DNA may be exploited (i) as a photochemical probe, allowing “photofootprinting” of G4 folding topologies *in vitro* and (ii) for covalent trapping of G4 structures as photoadducts with pyrene-substituted ligands.

## Introduction

Guanine-rich DNA and RNA sequences are capable of folding into non-canonic, four-stranded G-quadruplex (G4) structures, composed of several layers of guanine quartets that are stabilized by hydrogen bonds and coordination to central metal cations. G4 structures play important roles as regulatory elements in DNA- and RNA-related processes such as recombination, replication, transcription and genome maintenance^1,2^. Thus, recent bioinformatics^3,4^ and high throughput sequencing studies^5,6^ gave evidence of formation of numerous G4 structures by genomic DNA, with a particularly higher frequency observed in promoter regions of many genes, as well as in telomeres and in untranslated regions of mRNA^7,8^. However, the debate on the *in vivo* persistence and the exact roles of these structures continues^9,10^. Of note is the high polymorphism of G-quadruplexes which, unlike duplex DNA, can adopt very different topologies depending on their sequence and conditions. In spite of some attempts, the topology and stability of G4 structures can hardly be predicted purely on the basis of sequence information. In addition, a growing body of high-resolution structural data on G-quadruplexes gives evidence that these structures do not always follow the consensus motif (i.e., G_*n*_-N_*i*_-G_*n*_-N_*j*_-G_*n*_-N_*k*_-G_*n*_, where *n* = 2 to 4, *i*, *j*, *k* = 1 to 7, and N = any base)^11^. For example, snap-back G4 structures feature isolated guanine residues that participate in formation of G-quartets, leading to formation of complex folding topologies that can hardly be predicted by bioinformatics algorithms. In this context, experimental probing of formation of G4 structures in the genome, as well as their topology profiling, are still crucial for elucidation of persistence of these structures at various stages of the cell cycle and understanding their biological roles. Along these lines, reactive and photoreactive probes capable of forming covalent cross-links with G4 structures upon thermal^12–16^ or photochemical activation^17–19^, respectively, represent promising chemical biology tools for identification, isolation, and mapping of these motifs in the genome.

Complementary to ligand-induced photoreactions, photochemical probing of DNA using 5-halouracils as photoreactive analogues of thymine is a powerful method for characterization of protein–DNA interactions and local DNA conformations^20,21^. In this method, photo-induced dehalogenation of a 5-halouracil residue (usually ^Br^U or ^I^U) with UVB light (290–320 nm) yields a reactive uracil-5-yl radical, which subsequently induces atom-specific and highly conformation-dependent hydrogen abstraction reactions with sugar residues in the DNA backbone, leading to thermally labile 2′-deoxyribonolactone derivatives^22^ as well as intrastrand cross-links through reactions with neighboring purine bases^23,24^. Thus, systematic variation of the position of 5-halouracil residue within the DNA structure and the subsequent analysis of resulting photoproducts (i.e., the amount and the nature of the cleaved DNA strand and the cross-links) allows establishing the details of secondary structure of the DNA substrate. In the context of G4 DNA structures, Sugiyama *et al*. performed photochemical probing of ^I^U-modified G4-DNA formed by the human telomeric sequence, d[AGGG(TTAGGG)_3_] (hereafter 22AG), and demonstrated that only the antiparallel G4 structure formed in Na^+^ conditions (with ^I^U residues in the diagonal loop) efficiently underwent the photoreaction. In contrast, parallel or hybrid G4 structures formed in K^+^ conditions (i.e., devoid of diagonal loops) did not produce the 2′-deoxyribonolactone photoproduct upon UVB light irradiation^25^. A similar preferential reactivity of ^Br^U-modified diagonal loops was observed in the dimeric antiparallel G4 structure formed by the human telomeric repeat; notably, in the latter case, the ^Br^U residues located in the loop between the two G4 units was also highly reactive^26^. Another study demonstrated efficient formation of a d(G^U) crosslink in the ^Br^U-modified human telomeric sequence structure upon extended UVA light irradiation; importantly, the formation of the cross-link product was enhanced in Na^+^ conditions favoring formation of antiparallel G4 form with a ^Br^U residue in the second (diagonal) loop, compared to K^+^ conditions giving rise to a mixture of hybrid G4 isoforms^27^.

Besides direct photoexcitation of 5-halouracil residues with UVB light, generation of the uracil-5-yl radical may be achieved through an electron transfer reaction of ^Br^U with a photo-excited chromophore, typically pyrene, serving as electron donor, or “injector”^28^. Such ligand-sensitized photochemical probing is advantageous, since the chromophore can be selectively and efficiently excited with a lower-energy UVA light (e.g., λ = 365 nm for pyrene), thereby minimizing the non-specific DNA damage. In addition, ligand-sensitized electron injection can be correlated to the ligand binding site in the DNA structure (“photofootprinting”). This technique, developed by Sugiyama’s group, has been successfully employed for detection of binding sites of pyrene-conjugated pyrrole–imidazole polyamides, a family of sequence-specific minor-groove binders^29,30^.

**Figure 1:**
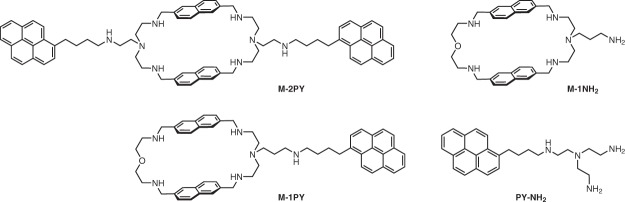
Structures of ligands studied in this work.

Herein, we studied ligand-sensitized photochemical reactions of ^Br^U-substituted G4-DNA structures belonging to different folding topologies (antiparallel, parallel, or hybrid). Towards this end, we employed two pyrene-substituted derivatives of the bis-naphthalene macrocycle 2,6-BisNP (hereafter M), namely M-1PY and M-2PY (Fig. 1)^31^, previously documented as affine and selective ligands for G4-DNA formed by the human telomeric sequence^32^. As control compounds, we employed the macrocycle M-1NH_2_ as well as a polyamine-substituted pyrene derivative, PY-NH_2_. Using gel sequencing experiments, we demonstrate that ligand-sensitized photoreactions of ^Br^U-substituted DNA are specific to G4 structures with respect to double-stranded and single-stranded (stem-loop) substrates. Moreover, using various DNA sequences, we show that these photoreactions may lead to different products (strand cleavage and covalent photoadduct), whose distribution is specific to the topology of the G4 substrate.

## Results

### Binding of ligands to various G4-DNA substrates

The affinity of ligands to various G4-DNA structures and selectivity with respect to double-stranded DNA was initially studied through fluorescence-monitored thermal denaturation (“FRET-melting”) experiments^33^. In this method, the extent of ligand-induced stabilization of G4-DNA is assessed by monitoring the denaturation of a G4-forming oligonucleotide terminally labeled with a fluorophore (F, 5′-FAM) and a quencher (T, 3′-TAMRA). Moreover, the drop of ligand-induced stabilization of G4-DNA observed in the presence of unlabeled double-stranded competitor (ds26) provides an additional information on the ligand selectivity. FRET-melting experiments were performed with several quadruplex-forming oligonucleotides representative of different folding topologies (Table 1): F-21G-T (hybrid in K^+^ and antiparallel in Na^+^ conditions), F-22CTA-T (anti-parallel), F-CEB25^WT^-T (parallel with a long propeller loop), F-CEB25^L111^-T (simple parallel), and F-Pu24T-T (snap-back parallel).

**Table 1:** Oligonucleotides used in this study. Thymine residues printed in bold were substituted with ^Br^U for photochemical studies. Residues participating in formation of G-quartets are underlined; duplex-forming regions are double-underlined. The same sequences double-labeled with 5′-FAM and 3′-TAMRA (abbreviated as “F-G4-T”) were used in FRET-melting experiments, except for 22AG; F-21G-T was used as a double-labeled analogue of 22AG in FRET-melting experiments^47^. The topology of G4 loops is given in square brackets: *p*, propeller; *l*, lateral; *d*, diagonal.

Acronym	Sequence (5′ → 3′)	Topology
22AG	AGGG**TT**AGGG**TT**AGGG**TT**AGGG	hybrid-1 [*pll*], hybrid-2 [*llp*] (in K^+^);^48–50^ antiparallel [*ldl*] (in Na^+^)^51,52^
21G	GGGTTAGGGTTAGGGTTAGGG	hybrid-1 [*pll*], hybrid-2 [*llp*] (in K^+^); antiparallel [*ldl*] (in Na^+^)^47^
bcl2Mid	GGGCGCGGGAGGAA**TT**GGGCGGG	hybrid-2 [*llp*, with a long *loop 2*]^46^
bcl2Mid^T20^	GGGCGCGGGAGGAA**TT**GGG**T**GGG	hybrid-2 [*llp*, with a long *loop 2*]
22CTA	AGGGC**T**AGGGC**T**AGGGC**T**AGGG	antiparallel [*lll*]^53^
hras-1	TCGGG**TT**GCGGGCGCAGGGCACGGGCG	antiparallel [*lll*]^54^
CEB25^WT^	AAGGG**T**GGG**T**G**T**AAG**T**G**T**GGG**T**GGGT	parallel [*ppp*, with a long *loop 2*]^55^
CEB25^L111^	AAGGG**T**GGG**T**GGG**T**GGGT	parallel [*ppp*]^56^
Pu24T	TGAGGG**T**GG**T**GAGGG**T**GGGGAAGG	snap-back parallel [*pppd*]^40^
Pu24T^T21^	TGAGGG**T**GG**T**GAGGG**T**GGGG**T**AGG	snap-back parallel [*pppd*]^40^
ds26		self-complementary duplex
hp29		stem-loop
hp29^MM^		stem-loop + two mismatches (italics)

**Figure 2:**
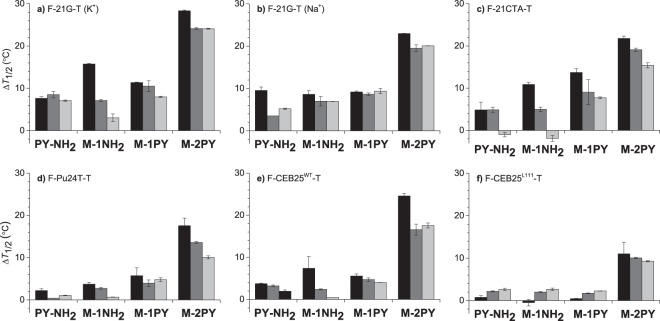
Ligand-induced stabilization of G4-DNA (Δ*T*_½_, °C) from FRET-melting experiments: (**a**) F-21G-T (K-10 buffer), (**b**) F-21G-T (Na-10 buffer), (**c**) F-21CTA-T (K-10 buffer), (**d**) F-Pu24T-T, (**e**) F-CEB25^WT^-T, and (**f**) F-CEB25^L111^-T (K-1 buffer), in the absence (black) or in the presence of 3 (gray) or 10 μM (light gray bars) of the duplex competitor (ds26). Conditions: *c*(F-G4-T) = 0.2 µM; *c*(ligand) = 1 µM; error bars represent the standard deviation from two experiments.

The results of FRET-melting experiments, presented as ligand-induced stabilization (∆*T*_½_), are presented in Fig. 2. Most importantly, M-2PY emerges as the most affine ligand of the series, with ∆*T*_½_ values of over 20–25 °C for all G4-DNA substrates, except for CEB25^L111^. For comparison, the benchmark ligand PhenDC_3_^34^ gave ∆*T*_½_ values of 28 to 31 °C in identical conditions (Supplementary Table S1). In the case of CEB25^L111^ (Fig. 2f), both M-2PY and PhenDC_3_ induced a lower stabilization effect (∆*T*_½_ = 11 and 13 °C, respectively; cf. Table S1), which can be attributed to the high intrinsic stability of this G4 structure (*T*_½_^0^ = 68.6 °C in the absence of ligands). In all cases, the stabilizing effect of M-2PY was only slightly reduced by the presence of the double-stranded competitor. Altogether, these data indicate strong and selective binding of this ligand to G4-DNA structures, with a significant preference to the hybrid structure formed by the human telomeric sequence (F-21G-T).

In identical conditions, M-1PY as well as control ligands M-1NH_2_ and PY-NH_2_ demonstrated much lower stabilization and lower selectivity for G4-DNA structures. Interestingly, the stabilization induced by M-1NH_2_ was, in most cases, comparable to that induced by M-1PY, indicative of similar affinity of both ligands for G4-DNA; however, the selectivity of the former was lower, as demonstrated by the significant drop of the ligand-induced stabilization in the presence of double-stranded competitor. This gives evidence that pyrene moiety is crucial for providing high selectivity for G4-DNA, as can be seen from rising selectivity in the series M-1NH_2_ < M-1PY < M-2PY.

**Figure 3:**
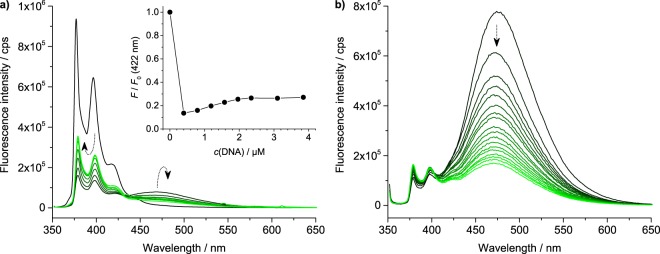
Spectrofluorimetric titrations of (**a**) M-1PY and (**b**) M-2PY (*c* = 2 µM in K-100 buffer) with 22AG. The dotted arrows as well as the inset in panel (a) show the changes of emission spectra of ligands upon addition of 22AG. Conditions: λ_ex_ = 347 nm, *c*(ligand) = 2 µM, *c*(22AG) = 0 (black) to 4 µM (green curve).

In parallel, the interaction of M-1PY and M-2PY with oligonucleotides was studied by means of fluorimetric titration experiments. In the case of M-1PY, titrations with a G4-DNA (22AG in K^+^ conditions) revealed a complex evolution of emission spectra, namely a dramatic decrease of intensity of pyrene monomer band (λ_em_ = 422 nm) upon addition of only 0.2 molar equiv. of ligand, accompanied by appearance of a broad, long-wavelength excimer band (λ_em_ ≈ 470 nm). Upon further addition of G4-DNA (up to 4 molar equiv.), the monomer emission band was partly recovered, while the excimer band decreased (Fig. 3a). This intricate behavior precluded the determination of binding constants of the interaction; however, the transient formation of the excimer band provides a strong evidence of formation of species with a 2:1 (ligand : G4-DNA) stoichiometry in the presence of excess of ligand. In the case of M-2PY, the evolution of emission spectra was less complex: the excimer band, initially dominating in fluorescence spectrum of the ligand, was gradually decreased upon addition of 22AG, while the intensity of the minor blue (monomer) band was only marginally affected (Fig. 3b). The fitting of titration isotherms (Supplementary Fig. S2) to the independent-site model allowed the determination of binding constants with a variety of DNA substrates, assuming a 2:1 binding stoichiometry (fitting to a 1:1 binding model was unsatisfactory in all cases). The results (Table 2) demonstrated the highest affinity of M-2PY to anti-parallel G4-DNA 22CTA (*K*_a_ = 3.9 × 10^6^ M^−1^), followed by hybrid G4-DNA substrates 22AG (in K^+^ conditions) and bcl2Mid (*K*_a_ = 1.8 × 10^6^ M^−1^ in both cases). The lowest affinity was observed with a parallel G4-DNA CEB25^L111^ and the self-complementary duplex ds26 (*K*_a_ = 0.54 and 0.48 × 10^6^ M^−1^, respectively). Notably, the results of fluorimetric titrations are in a good agreement with FRET-melting data, which demonstrate lowest stabilization of CEB25^L111^ (among all G4-DNA substrates) and good selectivity with respect to the double-stranded competitor, ds26.

**Table 2:** Binding constants (*K*_a_, in 10^6^ M^−1^) of M-2PY with selected oligonucleotides, obtained by fitting the fluorimetric titrations data to a 2:1 binding model.

22AG (K^+^)	22AG (Na^+^)	bcl2Mid	22CTA	hras-1	CEB25^WT^	CEB25^L111^	Pu24T	ds26
1.8	1.4	1.8	3.9	1.1	0.54	1.5	1.5	0.48

### Binding of ligands or ^Br^U modifications do not perturb the folding topology of G4-DNA

**Figure 4:**
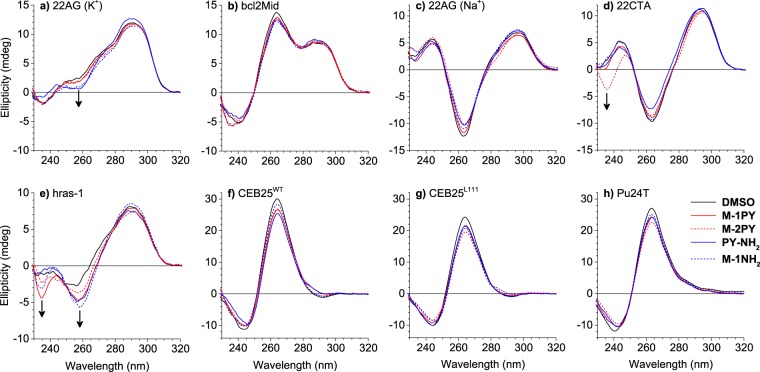
CD spectra of G4-DNA: (**a**) 22AG in K^+^ conditions, (**b**) bcl2Mid, (**c**) 22AG in Na^+^ conditions, (**d**) 22CTA, (**e**) hras-1, (**f**) CEB25^WT^, (**g**) CEB25^L111^ and (**h**) Pu24T, in the absence (DMSO control, black lines) and in the presence of ligands M-1PY (solid red), M-2PY (dashed red), PY-NH_2_ (solid blue) and M-1NH_2_ (dashed blue lines). The arrows indicate ligand-induced changes in CD spectra. Conditions: *c*(DNA) = 5 µM; *c*(ligand) = 10 µM in K-100 (**a**,**b**,**d**–**h**) or Na-100 (**c**) buffers.

Binding of ligands to G-quadruplex structures may induce conformation changes of the latter, as a consequence of a particularly high affinity of the ligand to one or another quadruplex fold. The most prominent example of this transformation is the shift from the hybrid conformation adopted by the human telomeric sequence (22AG) to an antiparallel form, induced by several potent ligands belonging to bisquinolinium series^35–37^. As circular dichroism (CD) spectra of G-quadruplexes are characteristic of their conformation^38^, we recorded CD spectra of several quadruplex-forming sequences used in this work in the absence and in the presence of two molar equivalents of ligands (Fig. 4). At the employed conditions (100 mM K^+^ or Na^+^), all sequences showed CD spectra typical of their topology (cf. Table 1). In the case of 22AG, the addition of M-1NH_2_ and PY-NH_2_ induced small changes in CD spectra obtained in K^+^ conditions, namely a decrease of CD signal at 260 nm, indicative of partial conversion from hybrid to antiparallel form (Fig. 4a); however, this change was not observed with the ligands of interest (M-1PY and M-2PY). In the case of hras-1, addition of all ligands led to a small increase of the negative band observed at 260 nm (Fig. 4e), giving evidence of further stiffening of the antiparallel fold, characteristic for this sequence. In the case of other sequences, none of the studied ligands induced significant changes in CD spectra in the spectral region characteristic of G4 folding topology (Fig. 4b–d,f–h). Thus, it can be assumed that the pyrene-substituted ligands M-1PY and M-2PY do not perturb the intrinsic conformations of G4 structures, and can be safely used as G4 topology probes.

We also assessed the impact of the T > ^Br^U substitution, required for photochemical studies, on the conformation of G4-DNA substrates. It has been previously observed that partial ^Br^U substitution in the human telomeric sequence (22AG) does not lead to conformation changes of the latter^25,27^; however, the consequences of a *total* substitution, as well as the impact of this modification on other G4-DNA structures, have not yet been examined. We recorded CD spectra of a number of ^Br^U-modified quadruplex-forming sequences, belonging to different folding topologies (Table 1), and compared them with the ones of their unmodified counterparts. In all cases, CD spectra of ^Br^U-modified and unmodified substrates were nearly identical (Supplementary Fig. S2), confirming that ^Br^U modification, as a rule, did not perturb the G4 conformation. Hence, ^Br^U-modified sequences represent an adequate model for studies of ligand-sensitized photoreactions with G4-DNA.

### Photoinduced reactions with ^Br^U-substituted G4-DNA: human telomeric sequence

**Figure 5:**
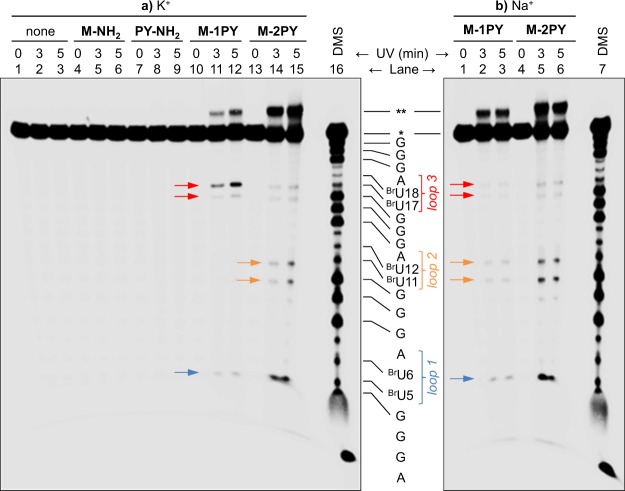
(**a**) PAGE analysis of ^Br^U-22AG irradiated in the presence of ligands (5 molar equiv.) in K^+^ conditions. Lane 1: control (non-irradiated) sample; lanes 2–3: ^Br^U-22AG irradiated in the absence of ligands; lanes 4, 7, 10, 13: ^Br^U-22AG incubated with ligands without UV irradiation; lanes 5–6: ^Br^U-22AG incubated with PY-NH_2_ and irradiated for 3 min and 5 min; lanes 8–9: ^Br^U-22AG incubated with M-1NH_2_ irradiated for 3 min and 5 min; lanes 11–12: ^Br^U-22AG incubated with M-1PY irradiated for 3 min and 5 min; lanes 14–15: ^Br^U-22AG incubated with M-2PY irradiated for 3 min and 5 min; lane 16: DMS/piperidine sequencing of ^Br^U-22AG. (**b**) PAGE analysis of ^Br^U-22AG irradiated in the presence of M-1PY (lanes 1–3) or M-2PY (lanes 4–6) in Na^+^ conditions; lane 7: DMS/piperidine sequencing of ^Br^U-22AG. Conditions used in both panels: *c*(DNA) = 5 µM, *c*(ligand) = 25 µM, irradiation with 365 nm UV light (300 W). Band assignment: *Unmodified DNA band; **Presumable covalent adduct. Colored arrows indicate the cleavage sites.

We performed photoreactions by irradiating mixtures of ^Br^U-22AG with ligands (5 molar equiv.) using UVA light (365 nm, 3 or 5 min) in K^+^ conditions (100 mM K^+^). After irradiation, samples were briefly heated to induce DNA cleavage at the presumably formed heat-labile sites and analyzed by denaturing polyacrylamide gel electrophoresis (Fig. 5a). Along with pyrene-containing ligands M-1PY and M-2PY, control compounds PY-NH_2_ and M-1NH_2_ were examined in order to elucidate the importance of each ligand moiety (i.e., polyazamacrocycle and pyrene) for the photochemical reaction. Importantly, in the absence of ligands, the ^Br^U-modified substrate was not affected by UVA irradiation (Fig. 5, lanes 1–3). Likewise, a sample of unmodified 22AG, irradiated in the presence of M-1PY or M-2PY in identical conditions, did not give rise to any photoproducts (Supplementary Fig. S3), giving a clear evidence that ^Br^U residues were indispensable for ligand-sensitized photoreactions.

According to our initial expectations, samples irradiated in the presence of M-1PY and M-2PY yielded a number of DNA strand cleavage products, evidenced as accelerated bands in PAGE analysis (Fig. 5a, lanes 11–12 and 14–15, respectively); in contrast, control compounds PY-NH_2_ and M-1NH_2_ did not yield detectable amount of cleavage products, clearly demonstrating the necessity of both parts of the ligand for photoinduced reactions. All photo-induced cleavage sites were located at 5′ ends of nucleotides flanking 5′ of the respective ^Br^U residues, as demonstrated through DMS/piperidine sequencing of a control sample of ^Br^U-22AG (Fig. 5, lane 16). This is in agreement with the cleavage mechanism involving pyrene-sensitized generation of uracil-5-yl radicals, followed by the formation of heat-labile 2′-deoxyribonolactone residues 5′ to ^Br^U residues (cf. Supplementary Fig. S4)^29,30,39^. Although a total yield of strand cleavage products obtained with M-1PY and M-2PY was comparable (5–7%), the distribution of cleavage sites revealed significant differences between these two ligands: for M-1PY, the principal cleavage sites originated from ^Br^U17 and ^Br^U18 (*loop* 3 of the G4 structure), whereas in the case of M-2PY most cleavage was due to ^Br^U residues of *loop 1* and *loop* 2.

Surprisingly, along with photoinduced DNA cleavage, we observed formation of a second kind of photoproducts, namely slower-migrating bands (** in Fig. 5), presumably corresponding to covalent adducts formed between ligands and DNA. These products were observed with M-1PY (~3% yield) and, particularly, M-2PY (~16%), but not in the absence of ligands or with model ligands (M-1NH_2_, PY-NH_2_). However, small amount of the slower-migrating product could be detected with PY-NH_2_ when used at a higher concentration (10 molar equiv., Supplementary Fig. S5); this gives an evidence that pyrene residues are involved in its formation. In a separate experiment, we assessed the influence of the ligand-to-DNA ratio and irradiation time on the yield of photoproducts. In the case of M-2PY, the formation of the slow-migrating photoadduct was observed already in the presence of stoichiometric amount of the ligand (~9%), and reached maximal yield (~20%, upon 5 min of irradiation) with ≥2 molar equiv. of M-2PY (Supplementary Figs S6 and S7). However, in the case of M-1PY, much higher amount of the ligand (10 equiv.) was needed to achieve a comparable yield of the photoadduct. This behavior is fully in line with the much higher G4-DNA affinity of M-2PY compared with M-1PY, observed in FRET-melting experiments, and speaks in favor of almost quantitative formation of a complex of the DNA substrate with M-2PY in our conditions.

We also observed that optimal yield of the photoproducts was obtained using short irradiation times (M-2PY: 3–5 min, M-1PY: 10–20 min). Upon longer UV exposure (30 min), the photoadduct was degraded, as demonstrated by the disappearance of the corresponding band in PAGE analysis (Supplementary Figs S6 and S7). This is consistent with the fact that, even in optimal conditions, the total yield of photoproducts does not exceed 30% (photoadduct: ~20%, strand cleavage: ~10%), presumably due to secondary photoprocesses such as photo-oxidation of the pyrene chromophore or light-induced degradation of primary photoproducts.

**Figure 6:**
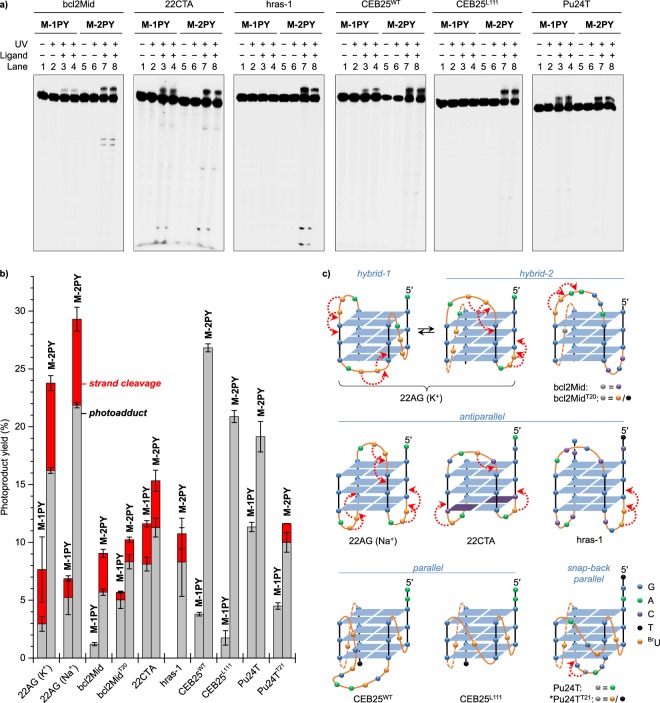
(**a**) PAGE analysis of ligand-induced photoreactions of ^Br^U-modified G4 structures: ^Br^U-bcl2Mid (hybrid-2), ^Br^U-22CTA (antiparallel), ^Br^U-hras-1 (antiparallel), ^Br^U-CEB25^WT^ (long-loop parallel), ^Br^U-CEB25^L111^ (parallel), and ^Br^U-Pu24T (snap-back parallel). Lanes 1 and 5: DNA only; lanes 2 and 6: DNA with UV; lanes 3 and 4: DNA and M-1PY with UV; lanes 7 and 8: DNA and M-2PY with UV (lanes 3 and 4, or 7 and 8, are the replica of identical experiments). Conditions as for Fig. 4; duration of UV exposition: 3 min. (**b**) Yield of photoproducts (red: DNA cleavage, gray: covalent adducts) formed in reactions of M-1PY and M-2PY with different ^Br^U-G4-DNA substrates (data from densitometric analysis of two independent experiments). (**c**) Structures of G4-DNA substrates used in this study and their susceptibility to ligand-sensitized photocleavage: the curly, dotted red arrows indicate the ^Br^U residues involved into strand cleavage and the respective cleavage sites.

In the quest of our understanding of ligand interaction with different isoforms 22AG, we also assessed the photoreaction of M-1PY and M-2PY with the antiparallel form of ^Br^U-22AG, predominantly formed in Na^+^ conditions (100 mM Na^+^). Similarly to what was observed in K^+^ conditions, both ligands gave rise to two kinds of photoproducts (Fig. 5b; quantification data: cf. Figure 6b). In the case of M-2PY, the yield of the covalent adduct was slightly higher in Na^+^ conditions (22%) comparing with K^+^ conditions (16%), while the one of cleavage products was not significantly affected (~7%). In the case of M-1PY, the yield of the photoadduct (~4%) was not significantly affected; however, the yield of cleavage products was reduced from 5% (in K^+^) to 2% (in Na^+^).

Next, we attempted to identify preferential reaction sites for photoinduced strand cleavage and covalent adduct formation with both ligands. For this purpose, the reactivity of each loop was systematically assessed by selectively substituting both thymine residues with ^Br^U, and the photoreactions were performed as described above. The results demonstrated no exclusive reactivity of any loop towards one or another photoreaction: in all cases, both kinds of photoproducts were observed (Supplementary Fig. S8). In the case of M-2PY, the *loop 1*-modified sequence (22AG^L1^) gave the highest amount of cleavage products both in K^+^ and in Na^+^ conditions, which is consistent with the distribution of cleavage products obtained with the all-^Br^U-substituted sequence (cf. Figure 5a). In the case of M-1PY, *loop 2*-modified sequence (22AG^L2^) gave the smallest yield of cleavage products (≤1%), with 22AG^L1^ and 22AG^L3^ giving comparable yields (3–5%). The formation of the covalent adduct was observed with all three ^Br^U-modified loops, with M-2PY giving systematically higher yields than M-1PY. Altogether, these results suggest that the topology of G4 substrate has a certain influence on the formation and distribution of photoproducts; however, the polymorphism of the human telomeric G4 structures makes the results difficult to interpret. Therefore, subsequent experiments were performed with other well-characterized G4 structures.

### Ligand-sensitized strand cleavage is specific with respect to loop topology in G4-DNA

In order to evaluate the impact of G4 topology on ligand-sensitized photoreactions, we exploited a panel of ^Br^U-modified analogues of other well-characterized G4-DNA structures (Table 1). Among these substrates, hybrid (bcl2Mid) and antiparallel (22CTA, hras-1) G4 folds gave rise to strand cleavage products upon photoreactions with M-2PY, with the cleavage sites corresponding to the distribution of ^Br^U residues in G4 loops (Fig. 6a). A lower total yield of ligand-sensitized strand cleavage (<5%, Fig. 6b), compared with 22AG, is likely due to a smaller number of reactive ^Br^U residues per substrate (two in bcl2Mid and hras-1, three in 22CTA, six in 22AG). In the case of M-1PY, strand cleavage could only be observed with 22CTA (~4%). In stark contrast, parallel (CEB25^WT^, CEB25^L111^) and snap-back parallel (Pu24T) G4 structures did not undergo strand cleavage in similar conditions (Fig. 6a). At the same time, the formation of slow-migrating covalent photoadducts was observed with all substrates. Quantification of gel electrophoresis data indicated that the adduct yield was systematically higher with M-2PY, compared with M-1PY, and with parallel G4-DNA substrates (up to 27% in the case of CEB25^WT^).

Taking into account the structural features of the G4 substrates (Fig. 6c), our observations may be rationalized upon assumption that ligand-sensitized DNA photocleavage is specific for ^Br^U residues of lateral and diagonal quadruplex loops. This model is consistent with the results obtained with 22AG, which forms two interconverting hybrid structures in K^+^ conditions: thus, photoinduced cleavage occurs in the lateral *loops 2* and 3 in the hybrid-1 structure, as well as in the lateral *loops 1* and *2* in the hybrid-2 structure; as a consequence, all three loops are cleaved in a sample containing a mixture of both forms. In Na^+^ conditions, the photocleavage affects the lateral *loops 1* and *3* as well as the diagonal *loop 2*. Likewise, 22CTA presenting three ^Br^U-containing lateral loops also gives rise to three distinct strand cleavage products. In the case of bcl2Mid and hras-1, each presenting two ^Br^U-residues in one of lateral loops, the cleavage is localized to these residues. In contrast, parallel folds CEB25^WT^ and CEB25^L111^ (which possess only propeller loops) as well as Pu24T (in which three ^Br^U residues are located in three propeller loops), do not yield any photocleavage products.

**Figure 7:**
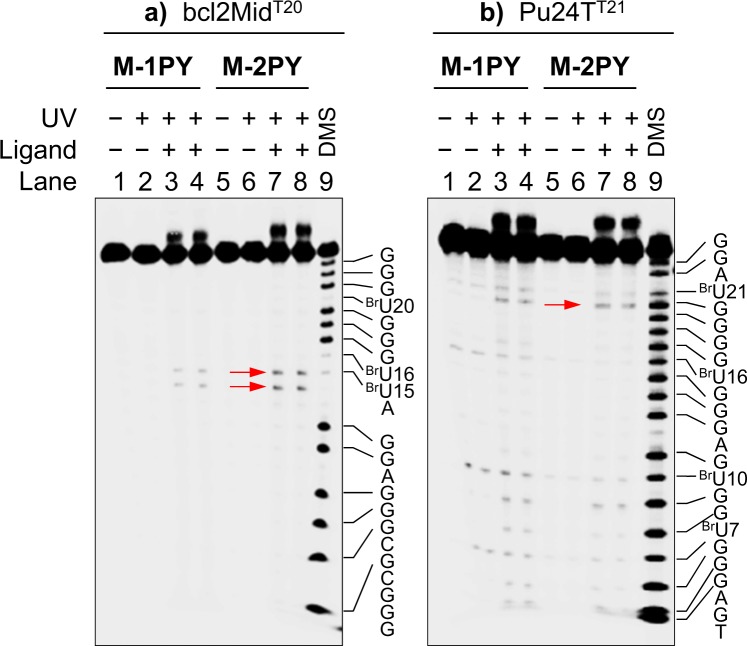
PAGE analysis of ligand-induced photoreactions of mutated, ^Br^U-modified G4 structures: (**a**) ^Br^U-bcl2Mid^T20^ (hybrid-2), (**b**) ^Br^U-Pu24T^T21^ (snap-back parallel). Lanes 1 and 5: DNA only; lanes 2 and 6: DNA with UV; lanes 3 and 4: DNA and M-1PY with UV; lanes 7 and 8: DNA and M-2PY with UV (lanes 3 and 4, or 7 and 8, are the replica of identical experiments); lane 9: DMS/piperidine sequencing. Conditions as for Fig. 4; duration of UV exposition: 3 min.

In order to confirm this hypothesis, we performed additional investigations with two hybrid structures offering interesting combinations of loops, namely bcl2Mid (hybrid-2, loop sequence: *llp*) and Pu24T (snap-back parallel fold presenting a forth diagonal loop in addition to the three propeller loops, *pppd*). In contrast to 22AG, these two structures, to the best of our knowledge, are not polymorphic and could allow the discrimination of propeller versus lateral/diagonal loops by ligand-sensitized photocleavage, provided the corresponding loops contain ^Br^U residues. However, while the T > ^Br^U substitution had no impact on the G4 topology, we anticipated that substitution of other bases could potentially disturb the intimate base–base interactions which define the G4 folding, and therefore had to be performed with great care. In the case of bcl2Mid, we deliberately replaced the cytosine C20 located in the propeller *loop 3* with a T (or a ^Br^U) residue (cf. Table 1 and Fig. 6c). In the case of Pu24T, we mutated the adenine residue A21 belonging to the diagonal *loop 4*; a detailed inspection of the Pu24T structure reveals that this adenine residue is not involved in hydrogen bonding interactions with other bases, and therefore may be replaced by another base without perturbing the overall folding motif^40^. The CD spectra of the two mutated sequences, bcl2Mid^T20^ and Pu24T^T21^, as well as their ^Br^U analogues, displayed only minimal differences with respect to the parent sequences (Supplementary Fig. S2c,j), confirming that their folding topology was not affected. Gratifyingly, ligand-sensitized photoreactions of these sequences provided full support of our hypothesis. Thus, the photoreaction pattern of ^Br^U-bcl2Mid^T20^ with M-1PY and M-2PY was identical to the one observed with ^Br^U-bcl2Mid (Fig. 7a), giving evidence that ^Br^U20, located in the propeller loop, does not give rise to photoinduced strand cleavage. On the other hand, a photoreaction of ^Br^U-Pu24T^T21^ with M-2PY resulted in a small, but significant yield (1.2%) of a strand cleavage product with a cleavage site localized 5′ to ^Br^U21 (Fig. 7b), confirming that this ^Br^U residue, located in the diagonal loop, is susceptible to the ligand-sensitized photocleavage. Altogether, these results demonstrate that ligand-sensitized photoreactions may discriminate the topology of different ^Br^U-containing loops within the same G4 structure, provided the substrate is not polymorphic in experimental conditions.

### Ligand-sensitized photoreactions are specific for G4 substrates

**Figure 8:**
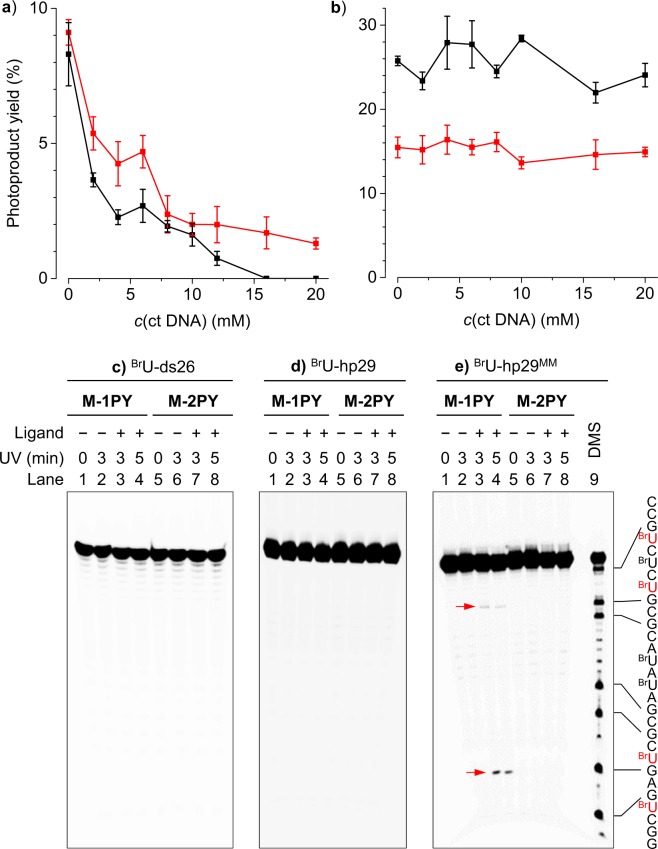
(**a**,**b**) Yield of products (red: DNA cleavage, black: covalent adduct) formed in photoreactions of ^Br^U-22AG (5 µM) with (**a**) M-1PY and (**b**) M-2PY (25 µM each) in the presence of ct DNA competitor, in K^+^ conditions. Error bars represent standards deviation from two independent experiments. (**c**–**e**) PAGE analysis of photoreaction of M-1PY or M-2PY with (**a**) self-complementary duplex ^Br^U-ds26; (**b**) stem-loop ^Br^U-hp29; (**c**) mismatch-containing ^Br^U-hp29^MM^ (the mismatched ^Br^U residues are typed in red). Lanes 1 and 5: only DNA; lanes 2 and 6: DNA with UV; lanes 3 and 4: DNA and M-1PY with UV; lanes 7 and 8: DNA and M-2PY with UV (365 nm, 3 and 5 min, respectively); lane 9 in (**e**) DMS/piperidine sequencing. Conditions: *c*(DNA) = 5 µM, *c*(ligand) = 25 µM in K-100 buffer.

To investigate the selectivity of ligand-sensitized photoreactions for G4 structures with respect to double-stranded DNA, we initially performed photoreactions with ^Br^U-22AG in the presence of increasing concentrations of calf thymus DNA (ct DNA) competitor. In the case of M-1PY, we observed a gradual decrease in the photoproduct yield upon increasing concentration of the competitor: thus, in the presence of 20 mM (phosphate) ct DNA, the yield of photoproducts was nearly zero (Fig. 8a and Supplementary Fig. S9). However, in the case of M-2PY, the yield of both photoproducts (i.e., cleaved DNA and covalent adduct) was not affected even at the highest used concentration of ct DNA (i.e., G4 DNA/ct DNA = 1:180). This result (Fig. 8b) suggests an exceptional selectivity of M-2PY for G4-DNA with respect to double-stranded DNA, and is in full agreement with the results of FRET-melting demonstrating high binding selectivity of this ligand.

Next, we assessed the *reactivity* of both ligands towards ^Br^U residues in various double-stranded and single-stranded DNA context. Taking into account the fact that bis-naphthalene macrocycles preferentially bind to the sites of pairing defects in double-stranded DNA, such as bulges and homopyrimidine mismatches^41,42^, one could reasonably expect enhanced photoreactivity at such sites. We studied the photoreactions of M-1PY and M-2PY with (i) a self-complementary 26-mer duplex DNA substrate (^Br^U-ds26, seven ^Br^U residues per strand); (ii) a hairpin structure with ^Br^U residues both in the stem and in the 5-nt loop parts (^Br^U-hp29); and (iii) an analogous hairpin bearing two ^Br^U<>^Br^U mismatches in the stem part (^Br^U-hp29^MM^, cf. Table 1). In the case of M-2PY, the analysis revealed no photoproduct formation with either of these substrates (Fig. 8c–e), giving a clear evidence that ligand-induced photoreactions are indeed specific for ^Br^U residues located in loops of G4 structures. However, in the case of M-1PY, we observed formation of a small amount (~2%) of strand cleavage products when the photoreaction was performed with the mismatch-containing substrate ^Br^U-hp29^MM^, with cleavage sites localizing to one of the mismatch sites (Fig. 8e). This result is in line with lower G4-DNA selectivity of M-1PY comparing with M-2PY, observed in other experiments.

### Insights into the nature of covalent photoadducts

**Figure 9:**
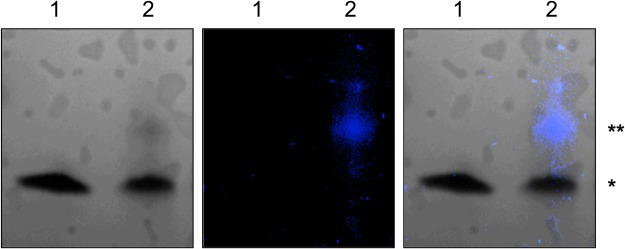
PAGE analysis of the products of a photoreaction of non-radioactively-labeled ^Br^U-22AG (50 µM) with M-2PY (125 µM) in K-100 buffer. Left: UV shadowing image of the electrophoresis gel under UV light (λ = 254 nm); middle: pseudo-color, auto-fluorescence image obtained with UV transillumination (λ = 305 nm); right: merged image. Lane 1: non-irradiated control, lane 2: photo-irradiated mixture (365 nm, 3 min); *Unmodified substrate, **Covalent adduct.

The slow-migrating products formed in photoreactions of ^Br^U-modified G4-DNA with M-2PY and M-1PY represent, most likely, covalent adducts formed between the substrate and the ligand, as was observed in related examples^17,19^. Indeed, a cross-link of two DNA strands would have a much lower mobility, as demonstrated by a control experiment with a 44-nt DNA fragment (Supplementary Fig. S10). Moreover, the fact that this product was systematically detected in samples that underwent thermal treatment (10 min at 95 °C, cf. Materials and Methods) speaks in favor of a stable, irreversible modification. To determine the nature of this modification, the product isolated from the slow-migrating band was subsequently treated with snake venom exonuclease, an enzyme which cleaves the DNA substrate in the 3′-to-5′ direction, stalling at crosslink and lesion sites^17^. While a control sample of ^Br^U-22AG was totally digested, the photoreaction product gave significant amount of unreacted material as well as two incomplete digest products, observed as slow-migrating bands in PAGE analysis (Supplementary Fig. S11). These results give additional evidence that the photoproduct represents an irreversible covalent modification (and not, for example, a non-covalent complex), which can be formed in three different sites, presumably corresponding to the ^Br^U-modified loops of the G4 substrate. Further evidence of formation of a covalent ligand–DNA crosslink was obtained through fluorescence analysis. Indeed, only the slow-migrating band demonstrated blue fluorescence under UV light (Fig. 9 and Supplementary Fig. S12). Moreover, DNA extracted from this band demonstrated a fluorescence spectrum with three well-resolved maxima at λ = 377, 397 and 419 nm, typical for pyrene derivatives (Supplementary Fig. S13). Altogether, these results provide an evidence that the covalent adduct is formed with the ligand (and not between two DNA strands) and contains at least one pyrene residue.

Lastly, we assessed the possibility of formation of a covalent adduct by performing a model photoreaction between PY-NH_2_ (*c* = 1 mM) and 5-bromo-2′-deoxyribouridine (BrdU, *c* = 10 mM) in an aqueous solution. In the absence of the template effect of G4-DNA, the photoreaction appeared notoriously inefficient despite the high concentration of reactants. However, after a prolonged UVA irradiation (365 nm, 3 h 30 min), LC/MS analysis revealed the formation of three novel, minor peaks characterized by UV absorption at 350 nm and identical *m*/*z* values of 629.5 (Supplementary Figs S14 and S15), presumably corresponding to covalent adducts forming in a reaction PY-NH_2_ with BrdU upon a loss of bromine atom. Although the structure of these adducts cannot be established at the current stage, the formation of three isomeric products is in line with the presence of several putative reactive sites in the structure of PY-NH_2_, most probably corresponding to α-carbons of amino groups. One plausible mechanism could involve hydrogen abstraction by uracil-5-yl radicals from α-carbon atoms of amino groups in the ligand, leading to generation of α-amine radicals and addition of the latter to nucleobases of DNA.

## Discussion

**Figure 10:**
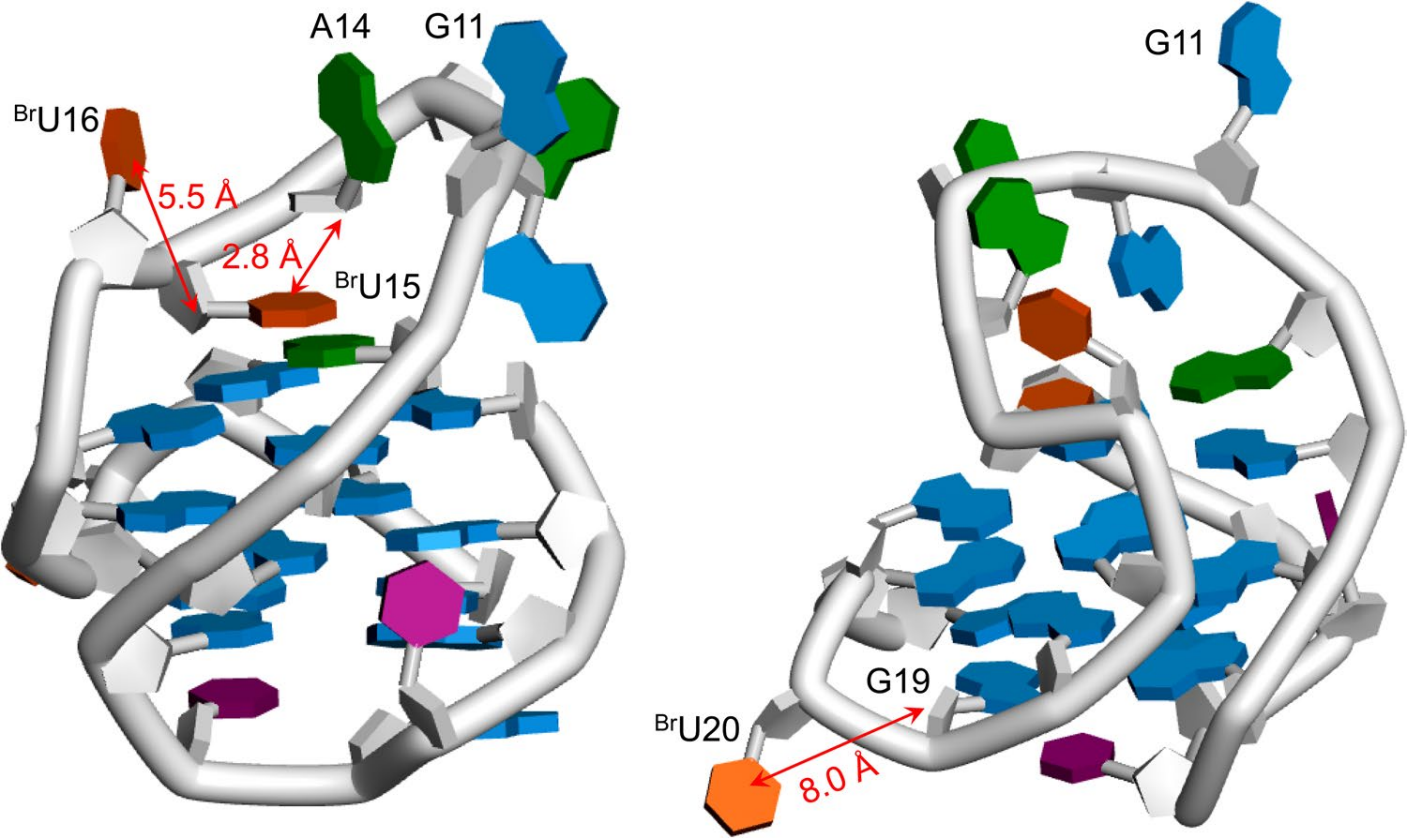
Putative structural model of ^Br^U-bcl2Mid^T20^ (based on the PDB entry 2F8U)^46^, illustrating the differences in photoreactivity of ^Br^U residues belonging to lateral (^Br^U15, ^Br^U16) and propeller (^Br^U20) loops. Nucleobase colors: G, blue; A, green, C, violet; ^Br^U, orange; the red arrows illustrate the distances between C5 of ^Br^U residues and H1′ of 5′-upstream nucleotides.

In the present work, we investigated the ligand-sensitized photochemical reactions of ^Br^U-modified G4-DNA belonging to different G-quadruplex folding topologies. We demonstrated that binding of both mono- (M-1PY) and bis-pyrene (M-2PY) polyazamacrocyclic ligands did not perturb the conformation of G4-DNA substrates, but induced photochemical reactions in ^Br^U-modified G4 loops. Notably, the bis-pyrene ligand systematically behaved as a significantly more efficient and G4-specific photosensitizer, as it induced no photoproduct formation with any of non-G4, ^Br^U-modified DNA substrates. The high efficiency and G4-specificity of M-2PY are likely due its higher binding affinity to G4-DNA (comparable with best benchmark G4-ligands), as reflected by FRET-melting experiments. Both ligands can induce two alternative photochemical reactions with ^Br^U-modified G4-DNA, namely DNA strand cleavage and formation of covalent photoadducts. Most spectacularly, the first process is observed exclusively with G4 structures presenting ^Br^U residues in diagonal or lateral loops, such as hybrid, antiparallel, or snap-back G4 folds. The loop-topology specificity of ligand-induced strand cleavage may be explained considering the structural differences of propeller and diagonal or lateral G4 loops, similarly to what was proposed by Sugiyama *et al*.^25^. Thus, ^Br^U residues of lateral or diagonal loops remain in proximity of neighboring nucleotides, allowing hydrogen abstraction from C1′ of 5′-flanking nucleotides upon photogeneration of uracil-5-yl radicals and the subsequent strand cleavage (Fig. 10, left panel). In contrast, ^Br^U residues of short propeller loops point outwards the G4 core and are remote with respect to C1′ of 5′-flanking nucleotides (cf. Fig. 10, right panel); consequently, hydrogen abstraction reactions leading to strand cleavage are highly unfavorable. Thus, ligand-sensitized strand cleavage may be considered as a sensitive photochemical probe for “photofootprinting” of G-quadruplex topology through discrimination of diagonal and/or lateral versus propeller loops.

Conversely, the second photochemical process, namely the formation of covalent ligand–DNA adducts, is observed with every studied G4 substrate, including those presenting ^Br^U residues in propeller loops. We may suggest that, in the absence of neighboring sugar residues predisposed for hydrogen abstraction reactions, the uracil-5-yl radicals of propeller loops, generated upon photoreaction with the pyrene-containing ligand, undergo a radical addition reaction with the same ligand molecule. Notably, the reactive species (uracil-5-yl radical) is generated on the G4 substrate and, in this sense, the formation of covalent adducts with pyrene-containing ligands described here is fundamentally different with respect to the previously described photochemical probes that covalently target G4-DNA through light-induced generation of *ligand-centered* reactive species, such as benzophenone diradicals, nitrenes^17,18^, or activated metal complexes^43,44^. Although the mechanistic details of this process could not be elucidated in details at the present stage, a preliminary support of such reaction could be provided by formation of photoadducts in a model photoreaction of BrdU with PY-NH_2_. Beyond the fundamental aspects shedding light on the reactivity of ^Br^U residues in the context of G4-DNA substrates, we may expect that this photochemical process could be exploited for design of novel agents for covalent trapping of G4 structures in longer sequences and potentially applied for identification of such structures in ^Br^U-labeled genomes. Thus, the bifunctional agents described in this work complement the currently available toolbox of photochemical tools for probing DNA structures.

## Materials and Methods

### Materials

Synthesis of M-1PY × 6 HCl, M-2PY × 6 HCl, M-1NH_2_ × 5 HCl and PY-NH_2_ was described previously^31^. Stock solutions of the ligands were prepared in a 1:1 (v/v) mixture of DMSO and H_2_O at a concentration of 1 mM. Oligonucleotides (purified by RP-HPLC for CD spectroscopy and FRET-melting experiments, or by gel electrophoresis for photochemical studies) were purchased from Eurogentec and solubilized in water at a concentration of 100 µM. All sequences were annealed in a suitable buffer prior to use, to allow folding of G4 structures, by heating at 95 °C for 5 min, followed by cooling in an ice bath for 1 h. Following buffers of identical ionic strength, but varied content of monovalent cations were used: K-100 (10 mM LiAsO_2_Me_2_, 100 mM KCl, pH 7.2), K-10 (10 mM LiAsO_2_Me_2_, 90 mM LiCl, 10 mM KCl, pH 7.2), K-1 (10 mM LiAsO_2_Me_2_, 99 mM LiCl, 1 mM KCl, pH 7.2), Na-100 (10 mM LiAsO_2_Me_2_, 100 mM NaCl, pH 7.2), and Na-10 (10 mM LiAsO_2_Me_2_, 90 mM LiCl, 10 mM NaCl, pH 7.2).

### FRET-melting

FRET-melting experiments were carried out with double-labeled G4-forming sequences F-21 G-T, F-21CTA-T, F-Pu24T-T, F-CEB25^WT^-T, and F-CEB25^L111^-T (Table 1), where F = 6-carboxyfluorescein (6-FAM) and T = 5-carboxytetramethylrhodamine (TAMRA). For analysis, 0.2 µM of pre-folded G4-DNA were mixed with 1 µM of ligand in absence or in presence of competitor DNA (self-complementary duplex ds26, 3 or 10 µM) in a corresponding buffer (total volume: 25 µL). Thermal denaturation runs were performed with a 7900HT Fast Real-Time qPCR instrument (Applied Biosystems) using a single heating ramp of 0.5 °C min^−1^ and monitoring the fluorescence intensity in the FAM channel.

### Fluorimetric titrations

Fluorescence emission spectra were recorded with a HORIBA Jobin-Yvon Fluoromax-3 spectrofluorimeter, using the excitation wavelength of 347 nm and slit widths of 2 nm. Titrations were performed by adding aliquots of pre-folded oligonucleotides (100 µM in K-100 or Na-100 buffers) to solutions of the ligands in the corresponding buffer (*c* = 2 µM) kept in a 1-mL cuvette. The binding isotherms, obtained by plotting fluorescence intensity at the emission maximum (474 nm for M-2PY) vs. concentration of added DNA, were fitted to the independent-site model as described elsewhere^45^.

### CD spectroscopy

Circular dichroism spectra were recorded with a J-710 spectropolarimeter (JASCO) equipped with a Peltier temperature controller, in quartz cells with a path length of 5 mm. Samples for CD spectroscopy contained 5 µM oligonucleotides in K-100 or Na-100 buffer, in the absence or in the presence of ligands (10 µM) or DMSO control (0.5% v/v). The scans were recorded at 20 °C from 210 to 330 nm using the following parameters: data pitch, 1 nm; bandwidth, 2 nm; response, 2 s; scan speed, 50 nm min^−1^; the reported spectra are the average of four scans.

### Photoreactions

Unless stated otherwise, samples were prepared by annealing a mixture of the 5′-^32^P-labeled G4-DNA (100 000 cpm) with 5 µM of the corresponding non-radiolabeled sequence to a total volume of 10 µL in K-100 buffer. The samples were incubated for 1 h at 37 °C in the presence of ligands (25 µM) and subsequently irradiated for 3 to 5 min with UV light (xenon light source MAX-303, 300 W, 365-nm monochromatic light) by keeping a distance of 10 cm between the light source and the sample in a 1.5-mL centrifuge tube placed on ice (irradiance: 5.44 mW cm^−2^). After irradiation, samples were concentrated in vacuo, supplemented with 10 µL of loading dye (bromophenol and xylene blue in a cocktail of 800 µL of formamide and 200 µL of 0.5 M EDTA)^39^, heated for 10 min at 95 °C to allow the cleavage of heat-labile 2′-deoxyribonolactone residues, and loaded onto the gel.

### DMS/piperidine sequencing

The 5′-^32^P-labeled substrate (50 000 cpm) was dissolved in 30 µL of water and incubated with 1 µL of dimethylsulfate (DMS) for 90 sec at 37 °C. It was then quenched by adding 90 µL of DMS stop buffer (1.5 M sodium acetate pH 7.0, 1 M β-mercaptoethanol, 100 µg mL^−1^ yeast tRNA) and precipitated by adding cold ethanol (900 µL). Finally, the DMS-treated DNA was incubated with 50 μL of 1 M aqueous piperidine solution for 25 min at 90 °C, inducing the cleavage at alkylated sites. After vacuum evaporation of piperidine, samples were washed four times with water and analyzed by 20% denaturing PAGE.

### Gel electrophoresis

Samples were analyzed by 20% denaturing PAGE (7 M urea, 1X TBE as running buffer) at 1000 V (run duration: 4 h). After exposure to a phosphor-imaging screen overnight, the gels were visualized with Typhoon 2000 scanner and quantified using ImageQuant software. The yield of photoproducts was estimated as the ratio of total counts of the photoproduct (crosslink or cleavage) bands to the sum of counts of photoproduct and unreacted DNA bands in the same lane, after background subtraction.

## Electronic supplementary material

Supplementary Information
