# Supplementary material for: Probing of G-Quadruplex Structures via Ligand-Sensitized Photochemical Reactions in BrU-Substituted DNA
Source: Sci Rep. 2018 Oct 25;8:15814. doi: 10.1038/s41598-018-34141-z (PMC6202380; doi:10.1038/s41598-018-34141-z)
Supplement: Supplementary file 1 — Supplementary Information [file 41598_2018_34141_MOESM1_ESM.pdf]

SUPPLEMENTARY INFORMATION

Probing of G-Quadruplex Structures via Ligand-Sensitized Photochemical Reactions in ^{Br}U-Substituted DNA

Abhijit Saha,[1,2] Sophie Bombard,[1,2] Anton Granzhan,*[,1,2] & Marie-Paule Teulade-Fichou*[,1,2]

[1] *CNRS UMR9187, INSERM U1196, Institut Curie, PSL Research University, 91450 Orsay, France*

[2] *CNRS UMR9187, INSERM U1196, Université Paris Sud, Université Paris Saclay, 91450 Orsay, France*

Table S1. Ligand-induced stabilization ($\Delta T_{½}$ / °C) of G4-DNA from FRET-melting experiments. [a]

**Ligand**	c_{ds26} / µM[b]	G4-DNA (F-G4-T)					
		22AG (K$^+$) [c]	22AG (Na$^+$)[d]	21CTA [c]	Pu24T [e]	CEB25^{WT} [e]	CEB25^{L111} [e,f]
**PY-NH$_2$**	0	7.6	9.5	4.8	2.2	3.7	0.7
	3	8.5	3.5	4.8	0.3	3.2	2.1
	10	7.1	5.1	−0.9	1.1	1.9	2.6
**M-1NH$_2$**	0	15.6	8.6	10.8	3.7	7.3	−0.4
	3	7.1	6.9	4.9	2.7	2.3	2.0
	10	3.1	6.9	−1.8	0.6	0.4	2.6
**M-1PY**	0	11.3	9.1	13.7	5.7	5.5	0.4
	3	10.5	8.6	9.0	3.9	4.7	1.7
	10	7.9	9.3	7.7	4.7	4.0	2.2
**M-2PY**	0	28.3	22.9	21.7	17.5	24.5	11.0
	3	24.1	19.5	19.1	13.6	16.5	10.0
	10	24.1	20.0	15.4	10.0	17.5	9.2
**PhenDC$_3$**	0	30.8	23.3	29.3	30.2	31.9	13.2
	3	32.6	23.4	28.9	28.4	31.0	16.8
	10	32.8	18.7	28.5	24.6	25.8	15.0
$T_{½}$ / °C [g]	0	57.5	57.7	61.9	56.2	54.4	68.5

[a] Conditions: $c_{F\text{-}G4\text{-}T}$ = 0.2 µM, c_{ligand} = 1 µM (except for CEB25^{L111}). [b] Concentration of the double-stranded competitor (ds26: Table 1). [c] In K-10 buffer. [d] In Na-10 buffer. [e] In K-1 buffer. [f] $c_{F\text{-}G4\text{-}T}$ = 0.2 µM, c_{ligand} = 0.2 µM. [g] Melting temperature in the absence of ligands.

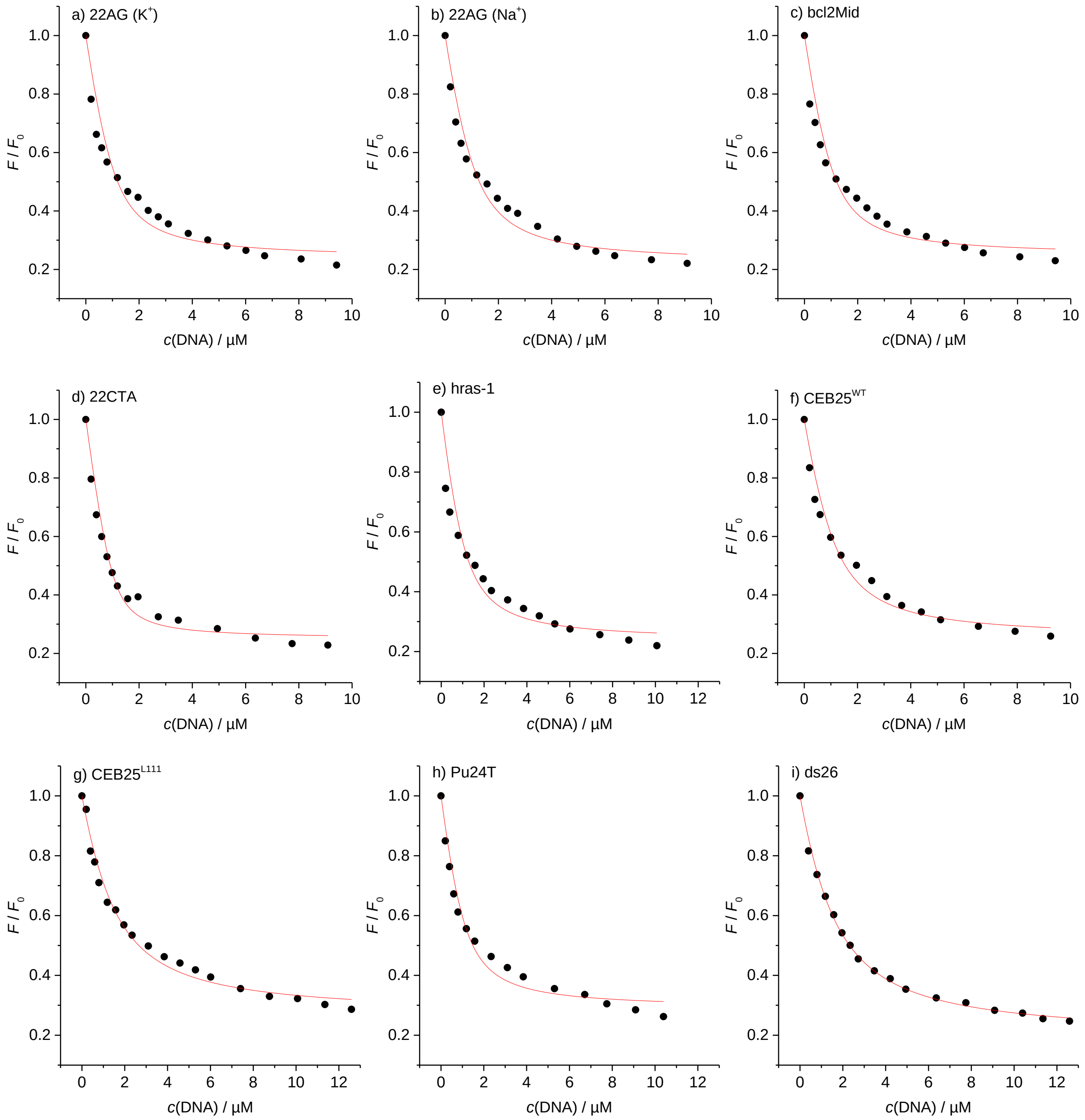

Figure S1. Binding isotherms from spectrofluorimetric titrations, presented as a relative change of the emission intensity of the excimer band of **M-2PY** (F/F_0, at λ_{em} = 474 nm) in the presence of various DNA substrates, as indicated in each panel. Red lines represent the fitting to the independent-site model using a 2:1 stoichiometry and binding constant (K_a) values given in Table 2. Conditions: *c*(**M-2PY**) = 2 µM in K-100 buffer (except for Na-100 buffer in panel b), excitation wavelength: 347 nm.

.

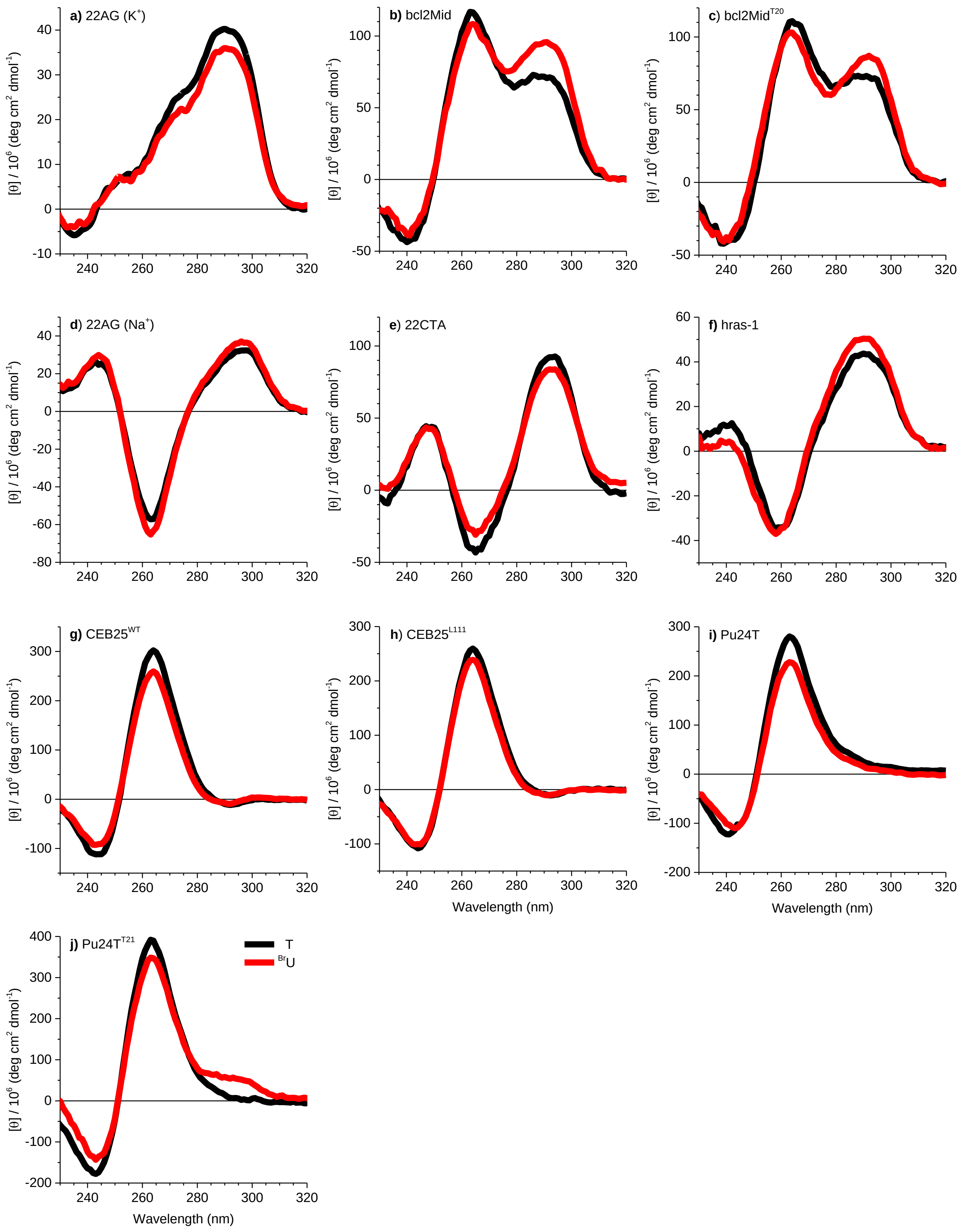

Figure S2. CD spectra of unmodified (black) and ^{Br}U-modified (red) G4-DNA structures (c = 10 µM in K-100 buffer, except for 22AG (Na$^+$): Na-100 buffer). The sequences are provided in Table 1.

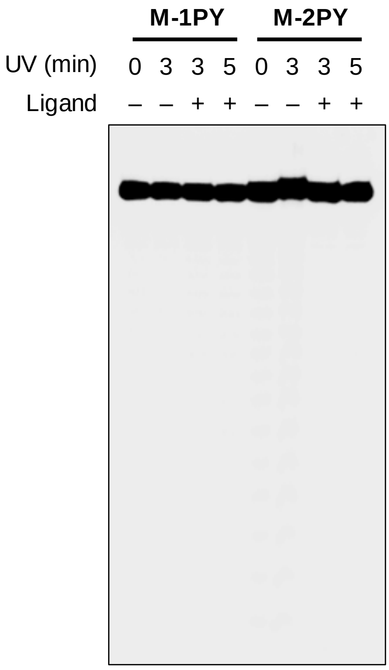

Figure S3. PAGE analysis of photo-irradiated reaction mixtures of unmodified 22AG with **M-1PY** and **M-2PY** in K^+ conditions. Conditions: *c*(DNA) = 5 µM, *c*(ligand) = 25 µM, irradiation with 365 nm UV light (300 W, 3 and 5 min).

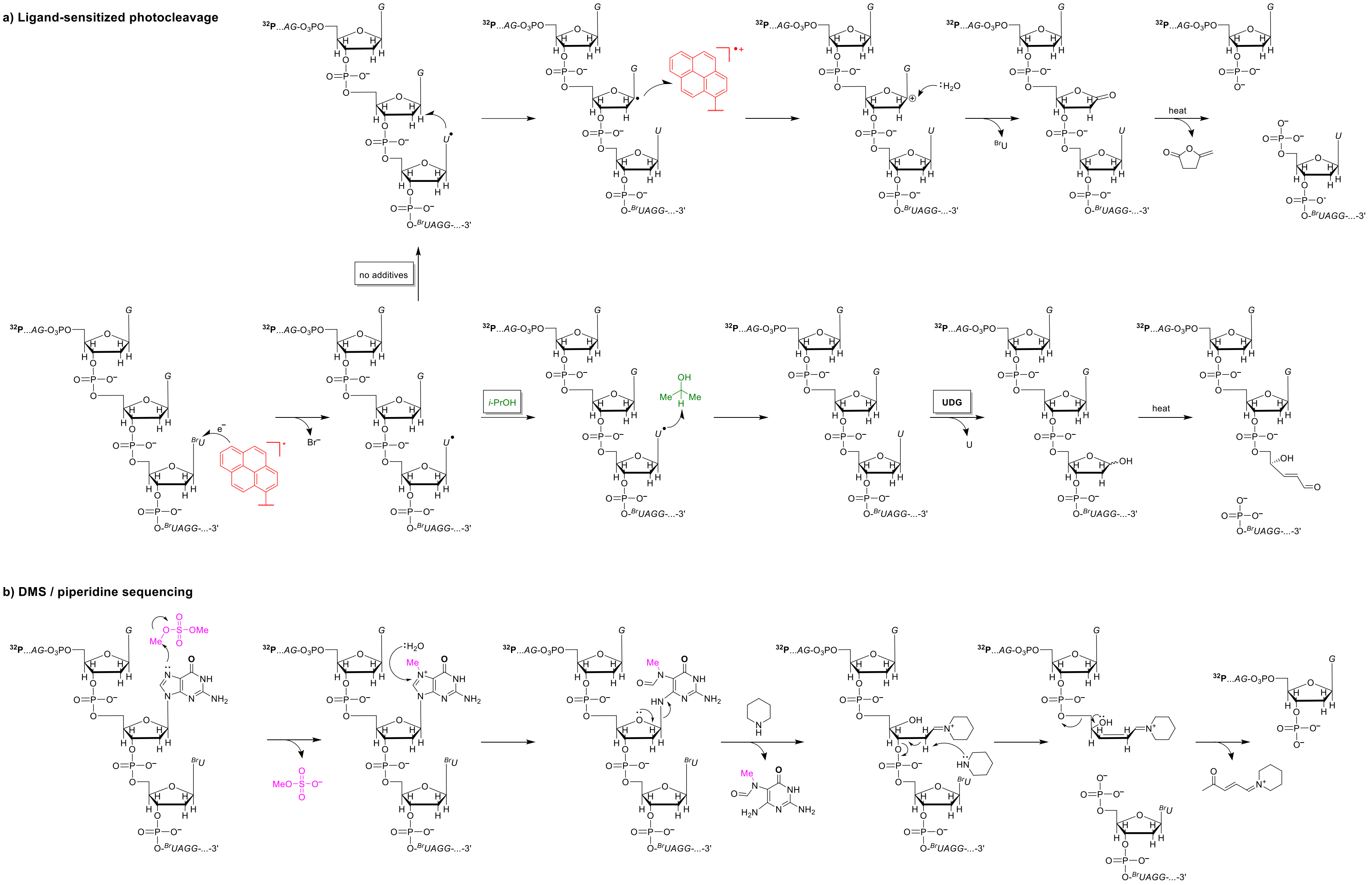

Figure S4. Reaction cascades corresponding to a) pyrene-sensitized photoclevage of ^{Br}U-modified DNA sequence (top row: no additives, bottom row: *i*-PrOH / UDG treatment) and b) DMS piperidine induced cleavage on ^{Br}U-modified G4-DNA. A fragment of the human telomeric repeat sequence (5′-…AGGG^{Br}U^{Br}UAGG…-3′) is shown in both cases, highlighting the difference in cleavage sites.

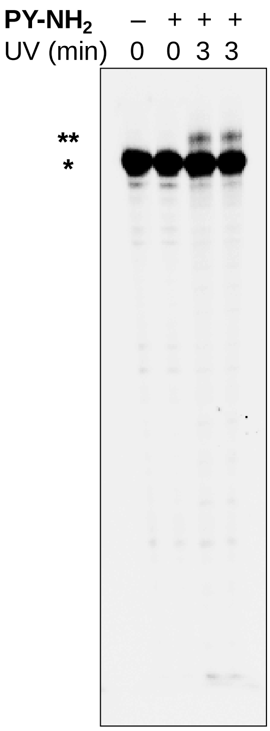

Figure S5. PAGE analysis of BrU-22AG irradiated in the presence of PY-NH2 (10 molar equiv.) in K^+ conditions. Conditions: *c*(BrU-22AG) = 5 µM in K-100 buffer, irradiation with 365 nm UV light (300 W). Band assignment: *, unmodified DNA band; **, presumable covalent adduct.

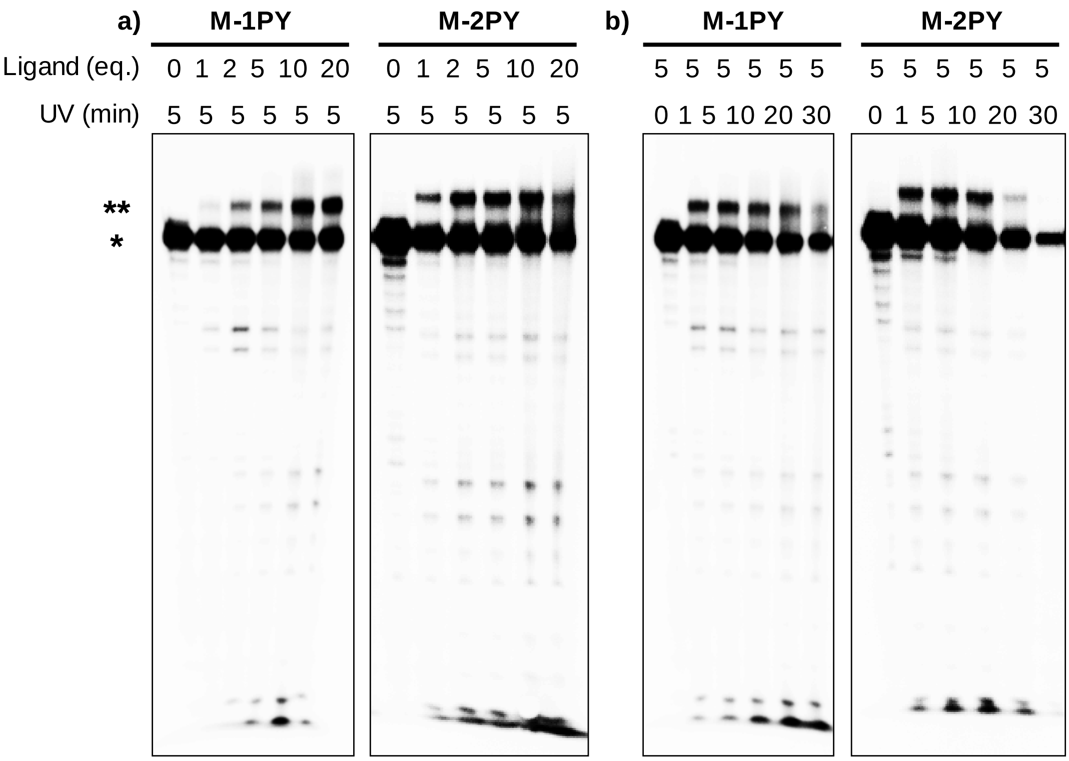

Figure S6. PAGE analysis of BrU-22AG irradiated in the presence of ligands **M-1PY** and **M-2PY**: a) using varied concentrations of the ligands; b) using different irradiation times, as indicated in the caption. Conditions: *c*(BrU-22AG) = 5 µM in K-100 buffer, irradiation with 365 nm UV light (300 W).

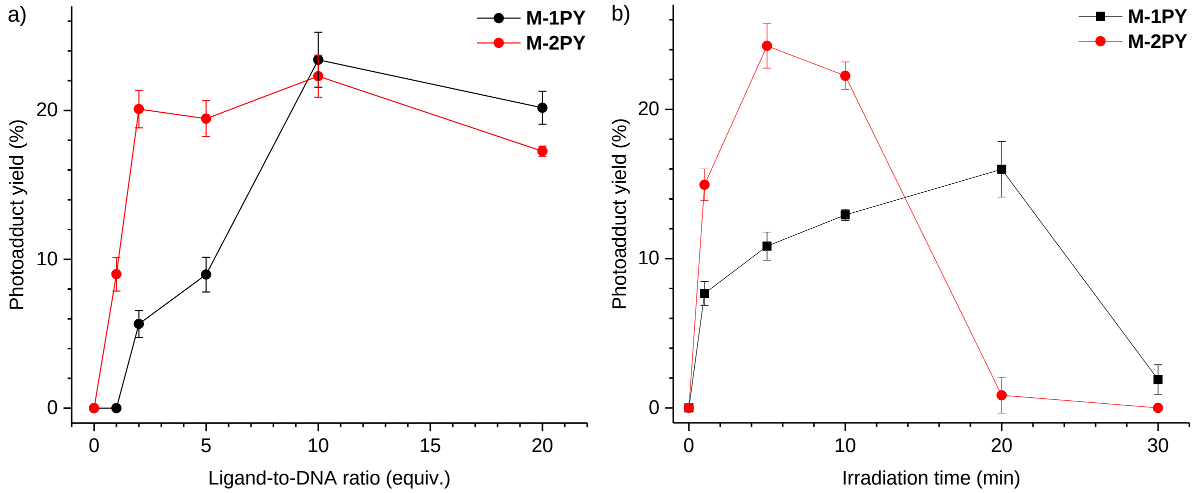

Figure S7. Influence of a) ligand-to-DNA ratio (using fixed irradiation time: 5 min, 365 nm) and b) irradiation time (using fixed ligand-to-DNA ratio: 5 molar equivalents) on the yield of the photoadducts (**) formed in ligand-sensitized photoreactions with ^{Br}U-22AG. Conditions as indicated for Figure S4. Data from densitometric analysis of gel electrophoresis data; error bars represent standard deviation from two independent experiments.

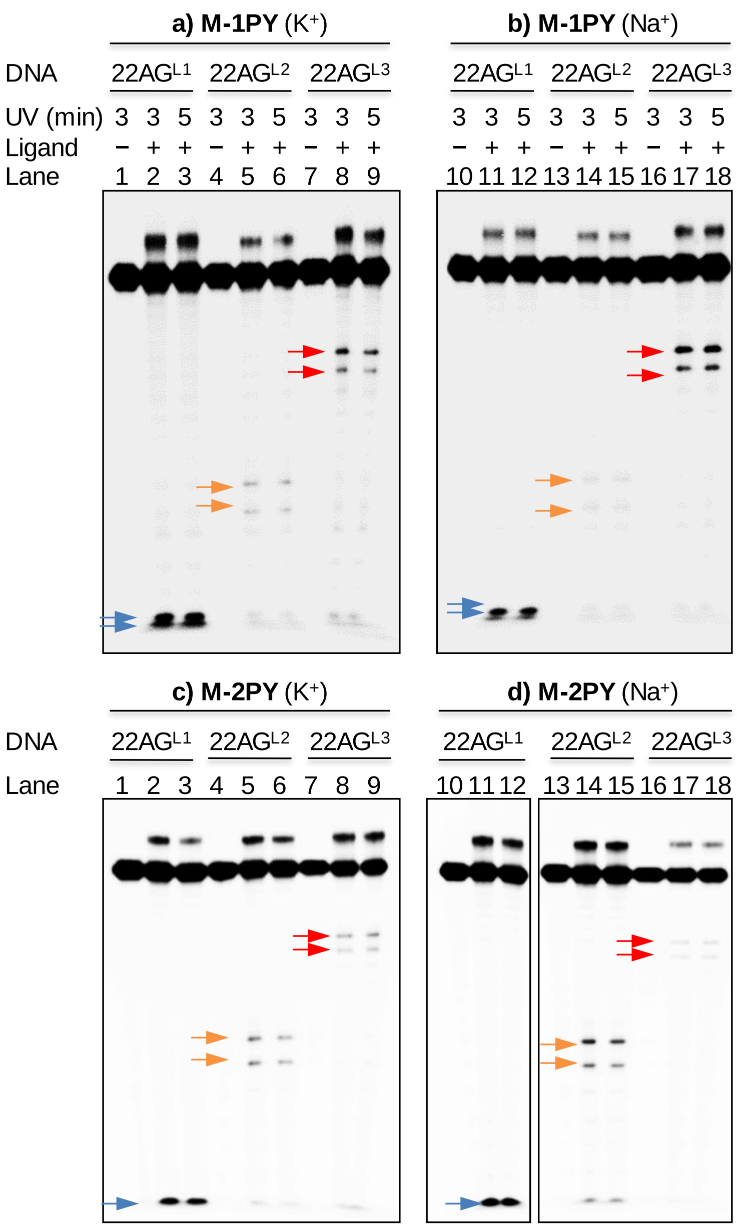

e)

22AG^{L1}: 5′-AGGG-^{Br}U^{Br}UA-GGG-TTA-GGG-TTA-GGG
22AG^{L2}: 5′-AGGG-TTA-GGG-^{Br}U^{Br}UA-GGG-TTA-GGG
22AG^{L3}: 5′-AGGG-TTA-GGG-TTA-GGG-^{Br}U^{Br}UA-GGG

Figure S8. PAGE analysis of photoreactivity of each loop of 22AG towards **M-1PY** (a–b) and **M-2PY** (b–c), assessed by variable substitution by ^{Br}U, both in K^+ (a, c) and in Na^+ (b, d) conditions. Conditions: *c*(DNA) = 5 µM, *c*(ligand) = 25 µM, irradiation with 365 nm UV light (300 W). Lanes 1, 4, 7, 10, 13, 16: UV-irradiated DNA controls, lanes 2, 3, 11 and 12: 22AG^{L1} irradiated in the presence of ligands, lanes 5, 6, 14, 15: same for 22AG^{L2}, lanes 8, 9, 17, 18: same for 22AG^{L3}. For the sequences of 22AG^{L1}, 22AG^{L2} and 22AG^{L3}, cf. Figure 4 in the main text.

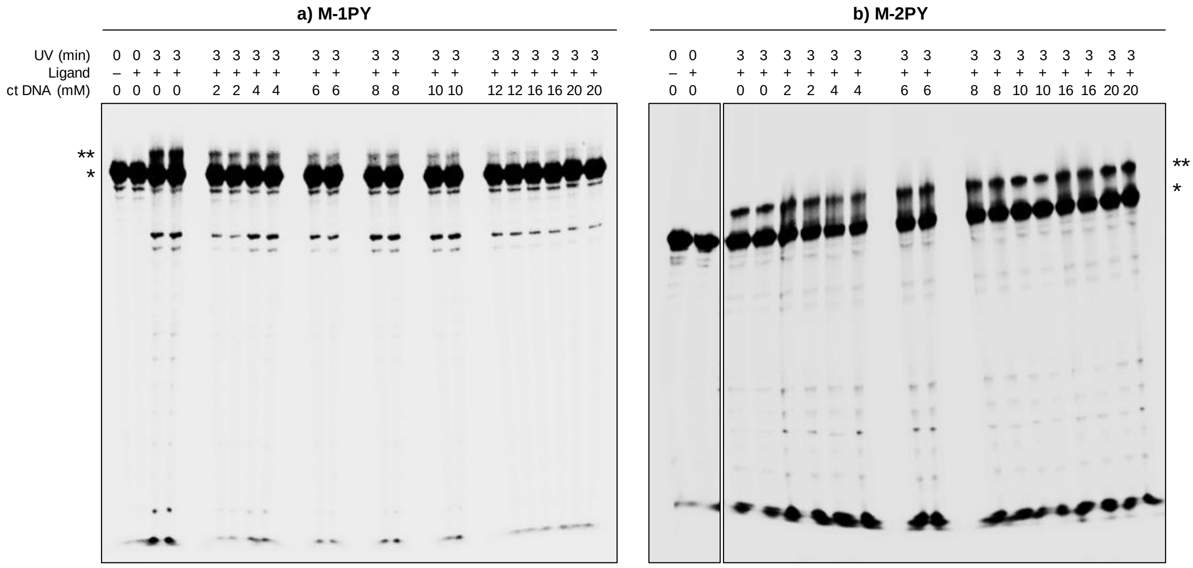

Figure S9. PAGE analysis of photoreaction of ^{Br}U-22AG (5 µM in K-100 buffer) with a) **M-1PY** and b) **M-2PY** (25 µM in each case) in the presence of increasing amounts of ct DNA competitor, as indicated in the header (0 to 20 mM). In panel b), both parts of the images are parts of the same gel. Quantification of these gels is shown in Figure 7 of the main text.

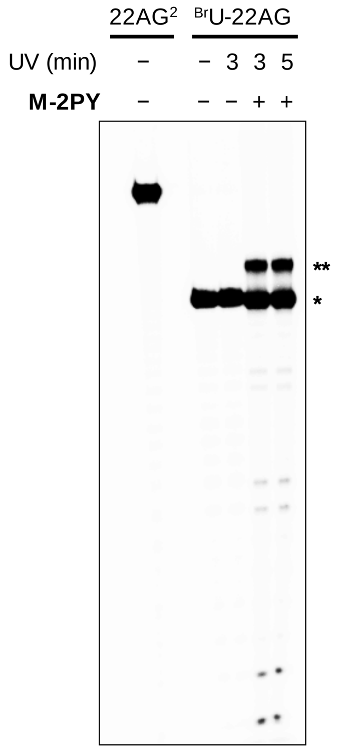

Figure S10. PAGE analysis of the products of a photoreaction of ^{Br}U-22AG with **M-2PY** and the comparison with a 44-nt oligonucleotide fragment [22AG^2: 5′-A(GGGTTA)$_3$GGGA(GGGTTA)$_3$GGG-3′]. *: Unmodified DNA band, **: presumable covalent adduct.

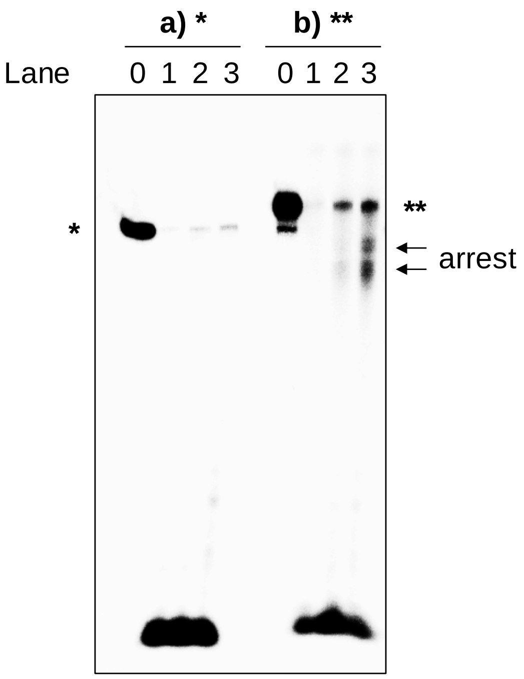

Figure S11. PAGE analysis of snake venom exonuclease treatment of unreacted ^{Br}U-22AG (*, panel a) and the putative covalent crosslinked product, formed in the photoreaction of ^{Br}U-22AG with **M-2PY** and excised from electrophoresis gel (**, panel b). Lane 0 in both panels: no exonuclease treatment, lane 1: treatment with 0.04 U, lane 2: 0.02 U, lane 3: 0.01 U of snake venom exonuclease in Tris-HCl, 5 mM $MgCl_2$ (pH 7.5) in the presence 0.5 mg mL^{-1} tRNA; incubation at 37 °C for 30 min.

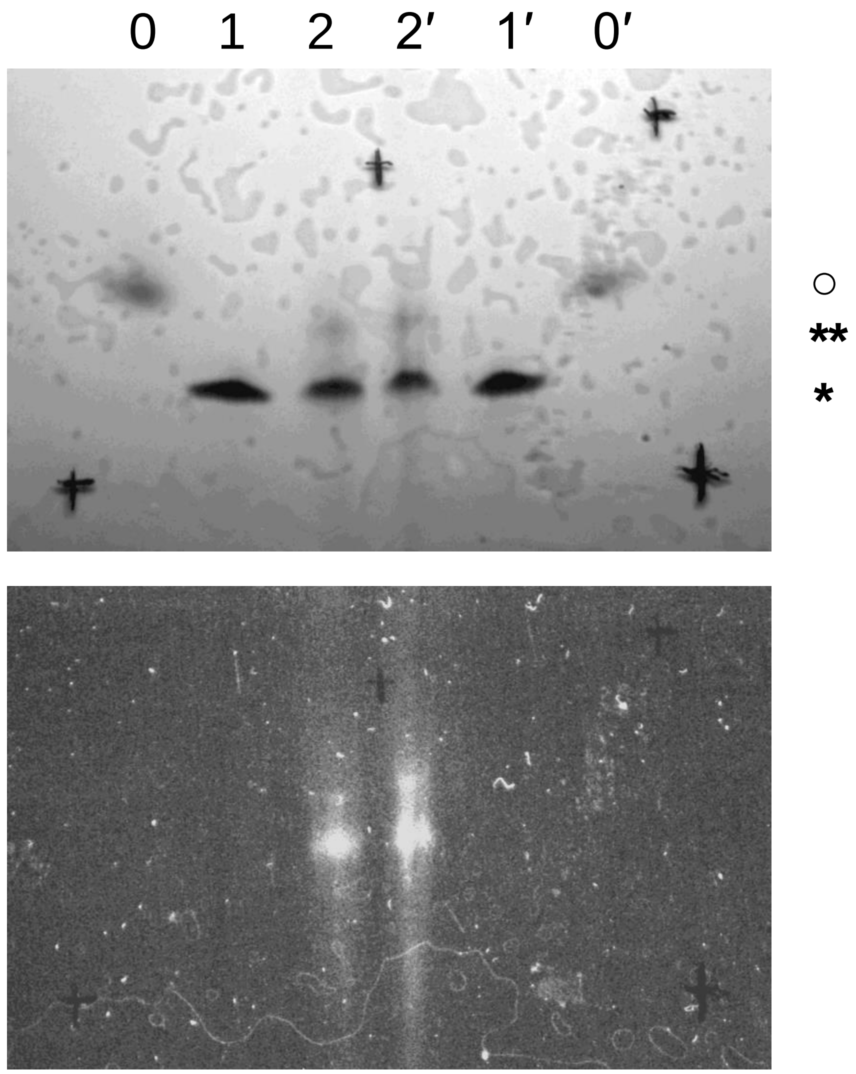

Figure S12. Full gel corresponding to Figure 9, a (with alignment marks). PAGE analysis of the products of a photoreaction of non-labeled ^{Br}U-22AG (50 µM) with **M-2PY** (125 µM). Top panel: UV shadowing image of the electrophoresis gel, obtained under UV light (λ = 254 nm) with the help of a TLC plate with F_{254} indicator. Bottom panel: auto-fluorescence image of the gel with UV transillumination (G:Box, λ = 305 nm). Lanes 0 and 0′: reference dye (Xylene Cyanol FF), lanes 1 and 1′: non-irradiated control, lanes 2 and 2′: photo-irradiated mixture (365 nm, 3 min). *: Unmodified substrate band; **: covalent adduct band; ○: Xylene Cyanol FF; **+**: reference marks used for image alignment.

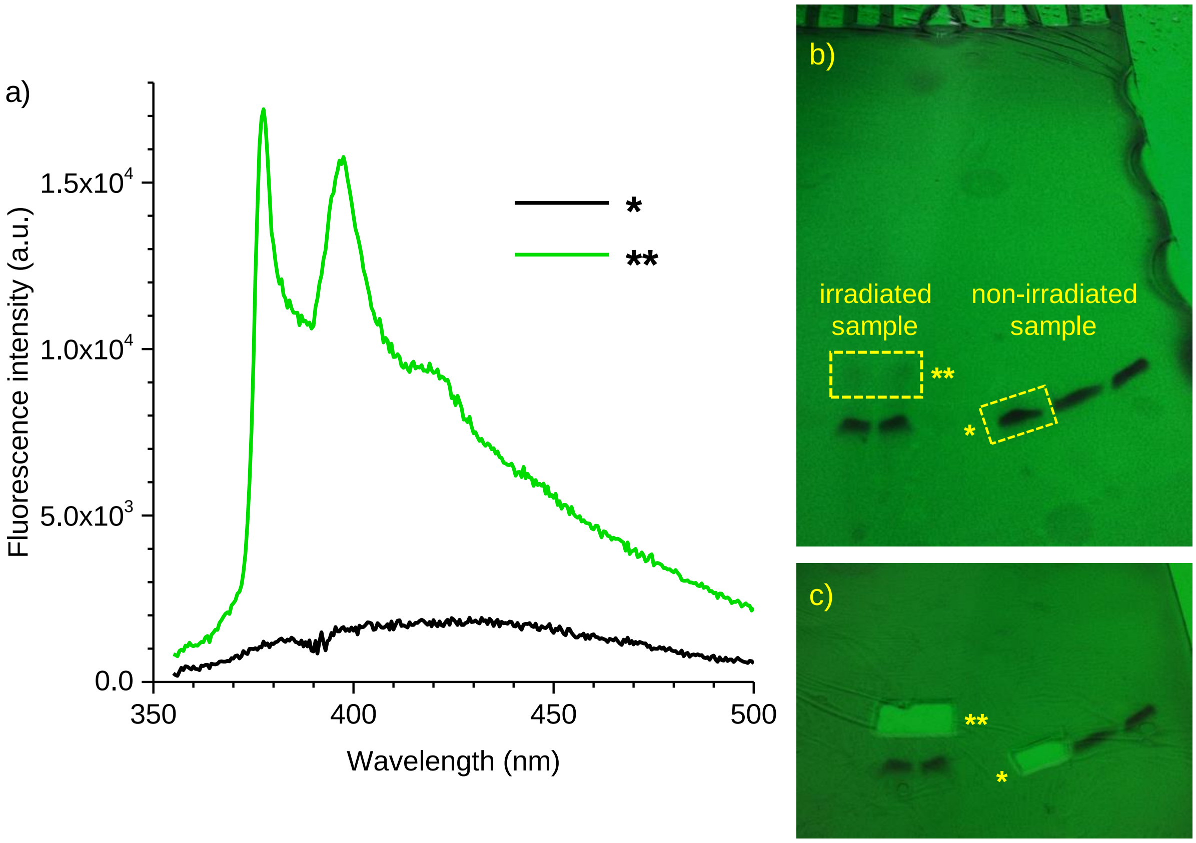

Figure S13. Fluorescence spectroscopy analysis of the photoproduct of reaction of ^{Br}U-22AG (50 µM) with **M-2PY** (125 µM). a) Background-corrected fluorescence emission spectra (λ_{ex} = 345 nm) of DNA fragments extracted from the electrophoresis gel: * control DNA band from the non-irradiated reaction mixture; ** slow-migrating band corresponding to the putative covalent ligand–DNA adduct. b–c) UV shadowing image of the electrophoresis gel, b) before and c) after cutting of the indicated bands (* and **). The image was obtained under UV light (λ_{ex} = 254 nm) using a TLC plate with an F_{254} indicator. The bands were cut, soaked in 500 µL of 0.15 M NaCl solution at 37 °C overnight, and the supernatant was analyzed by fluorescence spectroscopy.

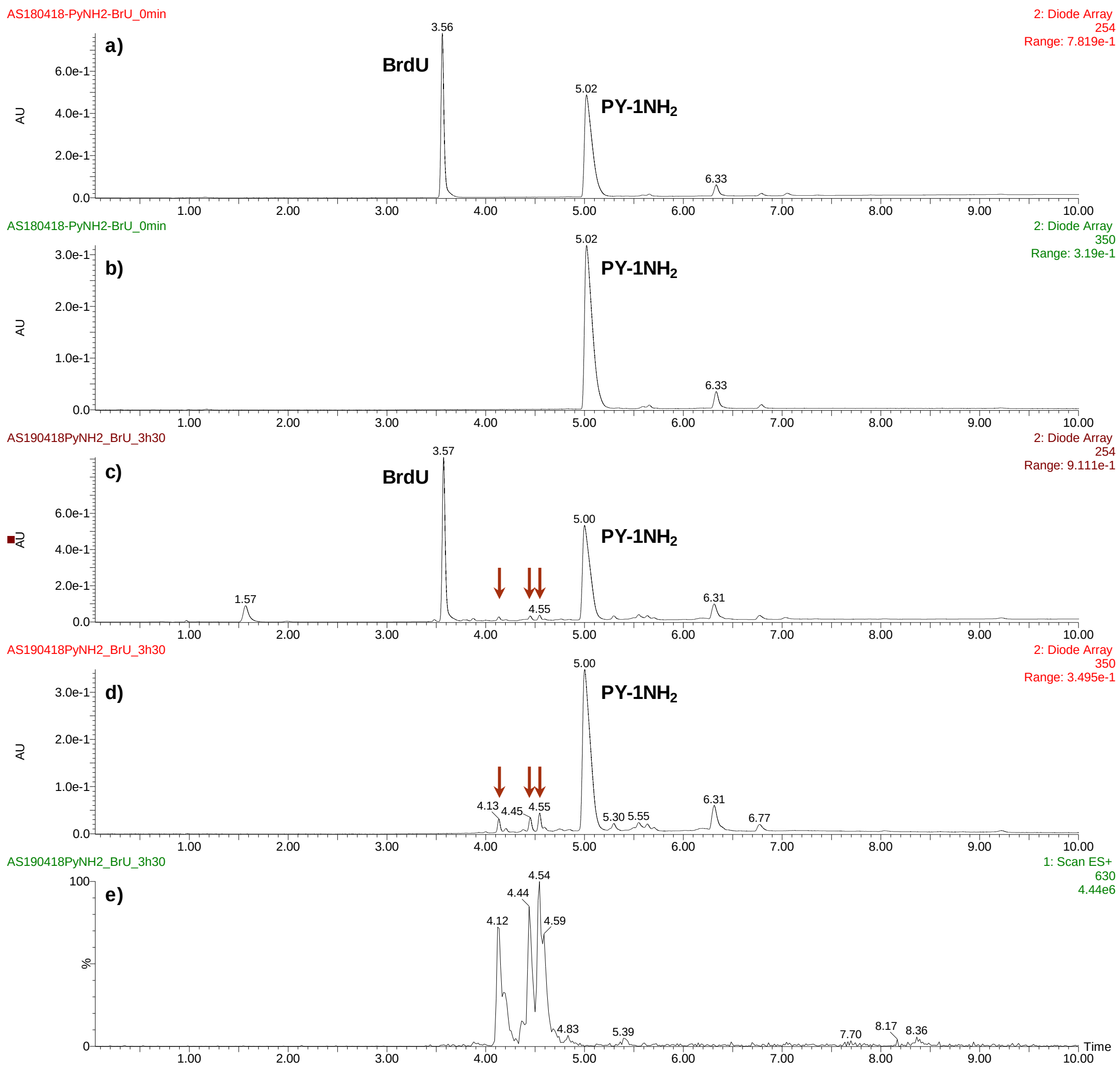

Figure S14. LC/MS chromatograms of a mixture of **PY-1NH$_2$** (1 mM) and BrdU (10 mM in 10 mM LiAsO$_2$Me$_2$ buffer, pH 7.2), before (a–b) and after (c–d) UV irradiation (365 nm, 3 h 30 min). Panels a) and c): single-wavelength chromatograms (λ = 254 nm); panels b) and d): single-wavelength chromatograms (λ = 350 nm); panel e): single-ion chromatogram (*m*/*z* = 630) corresponding to the expected covalent adducts. The red arrows indicate the putative covalent adduct peaks.

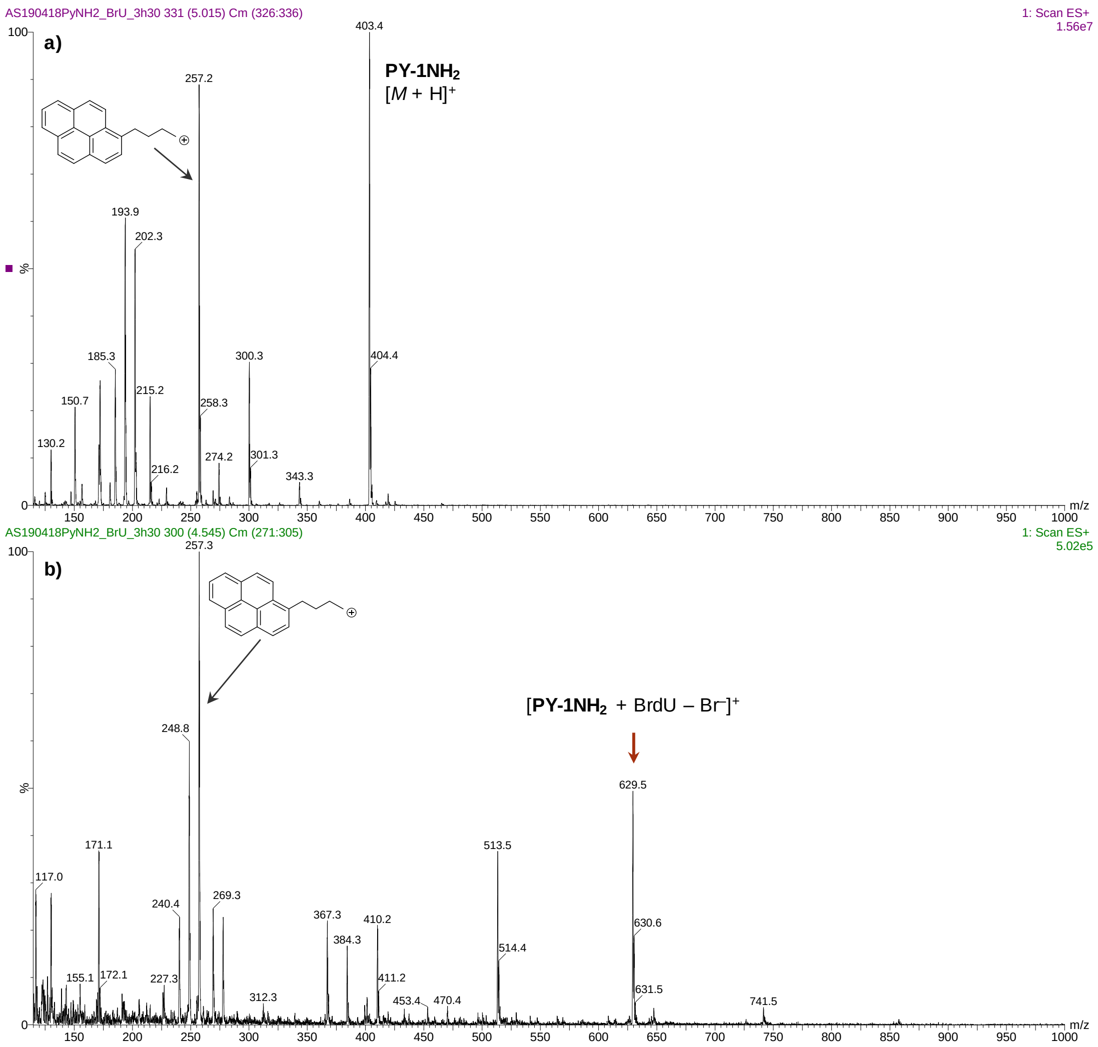

Figure S15. Mass spectra of peaks eluted at a) t_R = 5.0 min (**PY-1NH$_2$**, M = 402.6 g/mol) and b) 4.1–4.5 min (putative covalent adduct with BrdU, M = 628.8 g/mol).
